# Construction and characterization of an infectious cDNA clone of potato virus S developed from selected populations that survived genetic bottlenecks

**DOI:** 10.1186/s12985-019-1124-x

**Published:** 2019-02-06

**Authors:** Xin Li, Tatsuji Hataya

**Affiliations:** 1 Laboratory of Pathogen-Plant Interactions, Graduate School of Agriculture, Hokkaido University, Sapporo, 060-8589 Japan; 2 Laboratory of Pathogen-Plant Interactions, Research Faculty of Agriculture, Hokkaido University, Sapporo, 060-8589 Japan

**Keywords:** Potato virus S, *Carlavirus*, Infectious cDNA clone, Genetic bottlenecks, Quasispecies, Diversity, *Solanum tuberosum*

## Abstract

**Background:**

Infectious cDNA clones are a powerful tool for studies on RNA viruses using reverse genetics. Potato virus S (PVS) is a carlavirus with a worldwide distribution. Although the complete genome sequences of many PVS isolates have been reported, the construction of an infectious cDNA clone of PVS is yet to be reported. The aim of this study is the development and molecular characterization of an infectious cDNA clone of PVS.

**Methods:**

A full-length cDNA clone pPVS-H-FL-AB was constructed by connecting eight cDNA clones of PVS isolate H95. Capped RNA transcripts from pPVS-H-FL-AB and a modified clone pPVS-H-FL-H, containing the consensus genome sequence of PVS-H95, proved to be non-infectious. Therefore, a full-length cDNA clone pPVS-H-FL-β was reconstructed from PVS-H00, isolated from PVS-H95 populations by repeating a single local lesion isolation in *Chenopodium quinoa* three times; PVS-H00 appeared to be a selected variant that survived genetic bottlenecks. The sequence of cDNA clone pPVS-H-FL-β was determined as the genome sequence of PVS-H00 and compared with the consensus sequence of PVS-H95 genome.

**Results:**

All *Nicotiana occidentalis* plants inoculated with ≥0.2 μg capped RNA transcripts from pPVS-H-FL-β developed symptoms on upper leaves, as observed with PVS-H00 inoculation. Similar levels of viral genomic and subgenomic RNAs and coat protein were detected in systemically infected leaves. Sequence comparison of PVS-H95 and PVS-H00 revealed 370 nucleotide polymorphisms (4.4% of the entire genome sequence), causing 91 amino acid substitutions in six open reading frames (ORFs). The infectivity of chimeric RNAs derived from recombinants between the two cDNA clones revealed that the lack of infectivity of pPVS-H-FL-H transcripts was due to ORF1, which encodes replicase and harbors 80 amino acid substitutions compared with pPVS-H-FL-β. Approximately 71.3% amino acid substitutions in replicase were located within the variable region of unknown function between the putative methyltransferase and ovarian tumor-like protease domains.

**Conclusions:**

This is the first report of the development of an infectious cDNA clone of PVS. Our analyses suggest that PVS population within a plant exists as quasispecies and the replicase sequence diversity of PVS obstruct the construction of a full-length infectious cDNA clone.

**Electronic supplementary material:**

The online version of this article (10.1186/s12985-019-1124-x) contains supplementary material, which is available to authorized users.

## Background

Potato virus S (PVS) is one of the most common viruse of potato (*Solanum tuberosum* L.) with a worldwide distribution. PVS alone typically causes mild or no symptoms in most potato varieties; however, potato yield losses as high as 20% have been reported in the event of secondary infection [1, 2]. Two major biologically distinct strains of PVS, PVS^O^ (ordinary) and PVS^A^ (Andean), have been identified, based on the ability to systemically infect *Chenopodium quinoa* and *C. amaranticolor* plants. PVS^O^ induces chlorotic local lesions on the inoculated leaves of *C. quinoa* and *C. amaranticolor* without systemic infection, whereas PVS^A^ induces systemic chlorotic mottling in *C. quinoa* and *C. amaranticolor* plants after inducing chlorotic lesions on the inoculated leaves. In addition, PVS^A^ causes more severe symptoms on potato leaves, and it is more readily transmitted by aphids and contact with infected plants than PVS^O^ [3, 4]. Recently, however, some isolates that exhibit biological properties different from those of PVS^O^ and PVS^A^ strains have been reported from Tasmania, Australia [5]. Unlike PVS^O^, 13 out of 44 Tasmanian isolates cause local but asymptomatic infection only on inoculated leaves of *C. quinoa*. By contrast, nine isolates, unlike PVS^A^, cause systemic infection without the typical symptoms in *C. quinoa*. Of these nine isolates, five induce symptoms on inoculated leaves only, and four cause asymptomatic systemic infection on inoculated and uninoculated upper leaves [5].

Matoušek et al. [6] suggest that PVS-*Chenopodium-*systemic (CS) isolates from Central Europe are genetically distinct from PVS^A^ isolates, and they are closely related to European PVS^O^ isolates. Cox and Jones [7] proposed the term PVS^O-CS^ for isolates that systemically infect *Chenopodium* spp. but do not group within the PVS^A^ clade, according to phylogenetic analysis based on *coat protein* (*CP*) gene sequences. However, there is no evidence that CP is involved in systemic infection on *Chenopodium* spp. For instance, the *CP* gene sequence of Vltava isolate from Czech Republic groups within the PVS^A^ clade [7], although Vltava does not systemically infect *C. quinoa* [8]. Recombination analysis shows that the Vltava is a recombinant between PVS^O^ and PVS^A^ isolates [9]. Lin et al. [10] have reported that an isolate from the United States and two isolates from Chile fail to induce symptoms on the leaves of *C. quinoa*; phylogenetic analysis of *CP* gene sequences shows that the US isolate clusters with many PVS^O^ isolates, whereas both Chilean isolates cluster within the PVS^A^ clade [10].

To elucidate the genetic factors involved in the pathogenicity of PVS, e.g. systemic infection to *Chenopodium* spp., it is necessary to construct an infectious PVS cDNA clone; infectious clones are the most powerful genetic tool for reverse genetics of RNA viruses. Although the complete genome sequence of PVS was first reported in 2005 for a German isolate Leona [6], the construction of an infectious cDNA clone of PVS is yet to be reported.

PVS belongs to the genus *Carlavirus* [11], subfamily *Quinvirinae*, family *Betaflexiviridae*, and order *Tymovirales*. PVS particles are slightly flexuous filaments, with a length of ca. 650 nm and a width of ca. 12 nm [12]. The genome of PVS is single-stranded, positive-sense RNA, with a cap structure at the 5′ end and poly(A) tail at the 3′ end. The PVS genome is ca. 8.5 kb [6], with six open reading frames (ORFs). ORF1 occupies approximately 70% of the total genome length, and encodes a replicase protein of ca. 220 kDa containing the following five putative functional domains: methyltransferase (MTR), ovarian tumor-like protease (OTU-PRO), papain-like cysteine protease (P-PRO), RNA helicase (HEL), and RNA-dependent RNA polymerase (POL). The AlkB domain, which is involved in the direct reversal of alkylation damage, is found in some carlaviruses, such as blueberry scorch virus (BlScV) and hop latent virus (HpLV), but not in PVS or potato virus M (PVM) [13]. Of these five functional domains, one activity has been demonstrated in a carlavirus; P-PRO process the 223-kDa replicase between HEL and POL domains in BlScV autocatalytically [14]. Downstream of the replicase ORF are three overlapping ORFs, ORF2, ORF3, and ORF4, that together comprise a triple gene block (TGB), and encode proteins of ca. 25, 12, and 7 kDa, respectively, involved in cell-to-cell movement of the virus. ORF5 encodes the CP of ca. 34 kDa, and ORF6 encodes a cysteine-rich protein (CRP) of ca. 11 kDa that contains four cysteine residues in a specific consensus arrangement (Cys- X_2_-Cys - X_10–12_ - Cys - X_4_ - Cys) and possesses nucleic acid-binding properties [15]. The CRP of PVM, potato virus H, and sweet potato chlorotic fleck virus is reported to act as an RNA silencing suppressor [16–18].

Among carlaviruses, an infectious RNA transcribed in vitro from the full-length cDNA clone was first reported in BlScV in 1994 [19]. To date, the construction of an infectious full-length cDNA clone has been reported in a limited number of carlaviruses, including poplar mosaic virus [20], PVM [21], chrysanthemum virus B [22], cowpea mild mottle virus [23], and pea streak virus [24]. The full-length genome sequence of a Japanese isolate of PVS^O^ was determined in 2001 [25]. However, attempts to generate an infectious RNA transcribed from a full-length PVS cDNA clone in vitro have been unsuccessesful. Here, we report the successful construction and molecular characterization of an infectious full-length cDNA clone of PVS. Comparative genome sequence analyses suggest that quasispecies and the replicase sequence diversity of PVS make it difficult to construct an infectious full-length cDNA clone.

## Methods

### Virus isolates

The PVS isolate used in this study was originally detected on the potato cultivar Astarte in Hokkaido, Japan, in 1993 using enzyme-linked immunosorbent assay (ELISA). The virus was transmitted from a singly infected potato plant to *Nicotiana debneyi*, a systemic host of PVS, in 1995. PVS-H95 isolate was identified as an ordinary strain of PVS by reactivity on *C. quinoa*. Subsequently, *N. occidentalis* was identified as a symptomatic systemic host of PVS-H95, and determined to be suitable for the maintainance and purification of the virus. The full-length sequence of PVS-H95 genome extracted from purified virus particles was determined in 2001 [25]. Another isolate PVS-H00 was derived from a single local lesion on *C. quinoa* inoculated with PVS-H95 in 2000. In the meanwhile, the virus from the single local lesion on a *C. quinoa* leaf was propagated on *N. occidentalis* once and then inoculated on *C. quinoa* again. After three cycles of transmission from *C. quinoa* lesion to *N. occidentalis*, the isolate, designated as PVS-H00, was maintained on *N. occidentalis*. No biological and serological differences were observed between PVS-H95 and PVS-H00.

### RNA extraction, cDNA synthesis, and cloning

PVS-H95 genome was extracted from purified virions by proteinase K digestion, followed by phenol extraction and ethanol precipitation, as described previously [26] with some modifications. The RNA genome of PVS-H95 was converted to cDNA by reverse transcription-polymerase chain reaction (RT-PCR) using Moloney murine leukemia virus reverse transcriptase (Invitrogen, USA) and *Ex Taq* (Takara Bio, Japan). The long cDNA corresponding to most parts of ORF1 (ca. 5.4 kb) was amplified using a pair of PVS-11P and PVS-11 M primers (Additional file 1: Table S1) with a Takara RNA PCR kit (AMV) ver. 2.1 (Takara Bio, Japan). The RT-PCR products of the expected size were gel extracted using a MagExtractor PCR and Gel Clean-up Kit (Toyobo, Japan), and cloned into a pGEM-T Easy vector (Promega, USA) using T4 DNA ligase (Promega, USA).

**Fig. 1:**
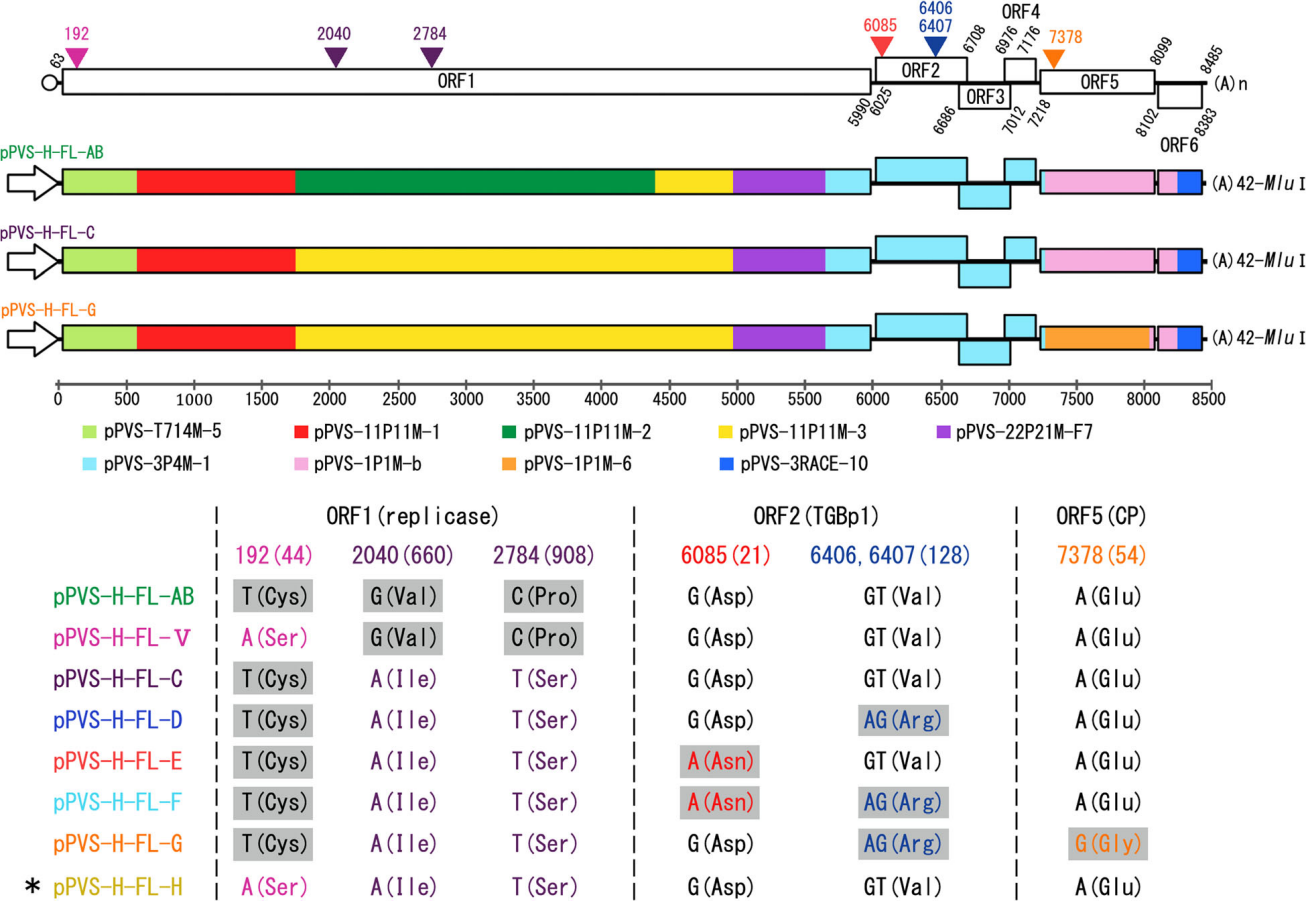
Schematic representation of the genome and full-length cDNA clones of PVS isolate H95. PVS-H95 genome organization is shown at the top. The start and stop positions of each ORF (rectangle) are indicated with nucleotide numbers. Nucleotide substitutions among the eight full-length cDNA clones of PVS-H95 (bottom) are indicated with colored arrowheads; numbers indicate the nucleotide positions. A full-length cDNA clone pPVS-H-FL-AB was constructed by connecting eight clones (colored rectangles). Arrow indicates T7 promoter. Seven additional clones were constructed either by the replacement of a fragment produced by site-directed mutagenesis (e.g., pPVS-H-FL-V, −D, −E, -F) or from a different clone (e.g., pPVS-H-FL-C, −G, -H). The cDNA clone pPVS-H-FL-H with an asterisk repesents the consensus sequence of PVS-H95 genome. Nucleotides and coding amino acids in parentheses with a gray background vary from the consensus sequence of PVS-H95 genome. Genomic sequence comparison between PVS-H95 (LC375227) and PVS-H00 (pPVS-H-FL-β, LC375228) is summarized in Table 2, Fig. 5 and Additional file 2: Figure S1

A full-length cDNA clone pPVS-H-FL-AB was constructed in a pNEB193 vector (New England Biolabs, USA) by connecting eight clones, as shown in Fig. 1. Of these eight clones, five were selected from clones used to determine the complete consensus genome sequence of PVS-H95, including pPVS-1P1M-b containing ORF5, pPVS-3P4M-1 containing ORF2–4, and pPVS-11P11M-3, − 2, and − 1 containing most of the ORF1 (Fig. 1). To construct pPVS-H-FL-AB, pPVS-3RACE-10 was first constructed by inserting a cDNA spanning viral 3′ terminal region synthesized by 3′RACE, as described previously [27]. To obtain a clone harboring a long poly(A) tail, timidin tail of an adaptor primer used in 3′RACE was elongated by terminal deoxynucleotidyl transferase (Takara Bio, Japan) before the RT reaction. In addition, a unique *Mlu*I restriction site was introduced immediately after the poly(A) tail comprising 42 adenine nucleotides (Fig. 1). Next, a cDNA clone pPVS-T714 M-5 was constructed by inserting a cDNA spanning viral 5′ terminal region fused to the bacteriophage T7 promoter produced by RT-PCR, after analysis of the 5′-terminal sequence of the PVS-H95 RNA genome by 5′ rapid amplification of cDNA ends (5′RACE) using FirstChoice RLM-RACE Kit (Ambion, USA) and by primer extension, as described previously [26]. Then, a cDNA clone pPVS-22P21M-F7 was constructed by inserting a cDNA spanning the POL coding region produced by RT-PCR.

The Ser44Cys substitution in replicase of pPVS-H-FL-AB was converted by site-directed mutagenesis, and pPVS-H-FL-V with Ser-44 was constructed (Fig. 1). The DNA fragment containing Ile660Val and Ser908Pro substitutions in the replicase ORF in pPVS-H-FL-AB was replaced by another clone pPVS-11P11M-3 to produce pPVS-H-FL-C with Ile-660 and Ser-908 (Fig. 1). Based on comparison with the other PVS genome sequences available from the DDBJ nucleotide sequence database, pPVS-H-FL-D, −E, and -F were constructed by the conversion of Val128Arg caused by GT6406-6407AG, Asp21Asn caused by G6085A, and both Val128Arg and Asp21Asn in TGBp1 encoded in ORF2 of pPVS-H-FL-C (Fig. 1). The *CP* gene fragment in pPVS-H-FL-D was replaced by the clone pPVS-1P1M-6 to produce pPVS-H-FL-G with Gly-54 (Fig. 1). Finally, Cys-44 in replicase of pPVS-H-FL-C was changed to Ser-44 by the replacement of cDNA fragment, resulting in the construction of pPVS-H-FL-H, whose sequence was identical to the consensus sequence of PVS-H95 genome (Fig. 1).

**Fig. 2:**
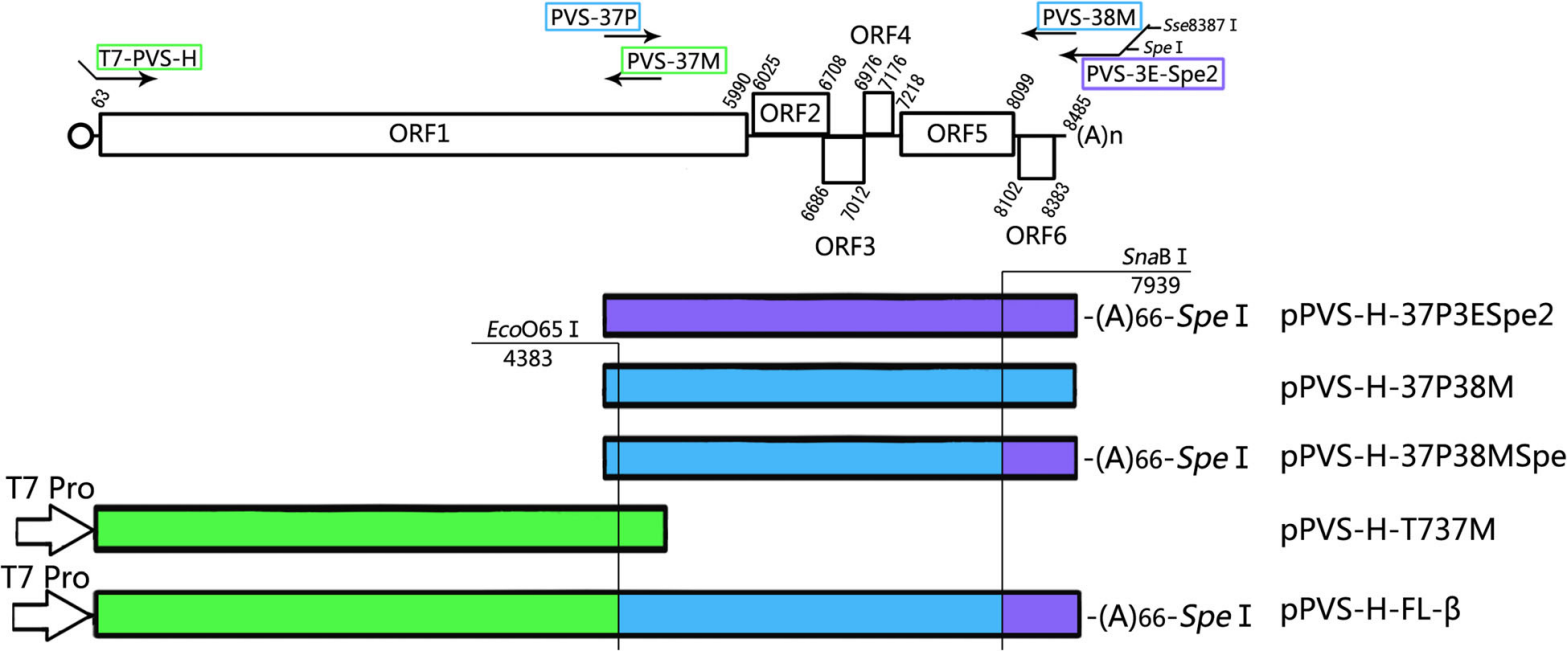
Schematic illustration of the construction of full-length cDNA clone of PVS-H00. Genome organization of PVS-H95 or PVS-H00 is shown at the top. The 3′ terminal half of PVS-H00 cDNA was amplified by PCR using PVS-37P and PVS-38 M as primers, and cloned to produce pPVS-37P38M. The 3′ terminal sequence downstream of the *Sna*BI restriction site in pPVS-H-37P38M was replaced with the 3′ terminal sequence containing a unique *Spe*I site immediately after the poly(A) tail of 66 adenine residues from pPVS-H-37P3ESpe2, which was a cDNA clone of PVS-H00. The 5′ terminal half of PVS-H00 cDNA was amplified by PCR using primers T7-PVS-H and PVS-37 M, and cloned to produce pPVS-T737 M. Finally, the 3′ terminal half of cDNA with the poly(A) tail and *Spe*I site from pPVS-H-37P38MSpe was connected with pPVS-T737 M via a unique *Eco*O65I site to produce the full-length cDNA clone pPVS-H-FL-β

A full-length cDNA clone was reconstructed using the PVS-H00 genome as a template. Total RNA was extracted using TRIzol Reagent (Invitrogen, USA) from dried leaves of *N. occidentalis* infected with PVS-H00, which were preserved after the single local lesion isolation in 2000. Two overlapping cDNA fragments were synthesized by RT-PCR using ReverTra Ace (Toyobo, Japan) and KOD-Plus- (Toyobo, Japan) with specific primers designed against the consensus sequence of PVS-H95 genome (Additional file 1: Table S1), as outlined in Fig. 2. The 5′ terminal half of the PVS cDNA (ca. 4.5 kb) was amplified using T7-PVS-H primer, homologous to the viral 5′ terminal sequence downstream of the T7 promoter sequence, and PVS-37 M primer. The 3′ terminal half of the PVS cDNA (ca. 4 kb) was amplified using the primer pair PVS-37P and PVS-38 M. Both RT-PCR products were gel purified, and cloned into a T-Vector pMD20 (TaKaRa Bio, Japan) by TA cloning to generate pPVS-T737 M and pPVS-37P38M (Fig. 2). The 3′ terminal sequence downstream of the *Sna*BI site in pPVS-37P38M was replaced with that from pPVSH-37P3ESpe2, which was constructed by cloning the 3′ terminal half of PVS-H00 cDNA into a pCR-Blunt vector (Invitrogen, USA), resulting in the introduction of a unique *Spe*I restriction site immediately after the poly(A) tail comprising 66 adenines at the 3′ terminus (Fig. 2). Finally, the 3′ terminal half of the cDNA with the poly(A) tail and *Spe*I site from pPVS-H-37P38MSpe was fused with pPVS-T737 M via a unique *Eco*O65I site, using T4 DNA ligase to construct the full-length cDNA clone pPVS-H-FL-β (Fig. 2).

Replacement of the cDNA fragment was performed at the restriction enzyme digestion sites using T4 DNA ligase. Nucleotide substitutions were performed using a QuikChange II XL Site-Directed Mutagenesis Kit (Stratagene, USA), or according to the protocol of KOD-Plus-Mutagenesis Kit (Toyobo, Japan).

### In vitro transcription and plant inoculation

The plasmid containing full-length PVS cDNA was linearized with *Mlu*I or *Spe*I for transcription. Digested DNA was purified by phenol/chloroform extraction and ethanol precipitation. Capped RNA was transcribed in a 20 μL reaction mixture, containing 40 mM Tris-HCl (pH 8.0), 25 mM NaCl, 8 mM MgCl_2_, 2 mM spermidine, 10 mM dithiothreitol, 2 mM each of ATP, UTP, and CTP, 0.2 mM GTP, 0.5 mM cap analog m^7^G(5′)ppp(5′)G (Invitrogen, USA), 40 U of human placenta RNase inhibitor (Wako, Japan), 5 μg of linearized plasmid DNA, and 50 U of T7 RNA polymerase (Invitrogen, USA), at 37 °C for 25 min. Subsequently, 2 μL of 20 mM GTP was added to the reaction to a final concentration of 2 mM. After 50 min incubation at 37 °C, plasmid DNA template was digested with 5 U of RNase-free DNase I (Takara Bio, Japan) at 37 °C for 15 min. The reaction volume was then increased to 100 μL with ultrapure water, and 10 μL of a solution containing 5 M ammonium acetate and 0.1 M EDTA (pH 8.0) solution was added to the reaction. The reaction products were purified by phenol/chloroform extraction, followed by precipitation with 1 volume of 2-propanol. The products were resuspended in THE RNA Storage Solution (Ambion, USA) containing 1 mM sodium citrate (pH 6.4). The RNA yield was estimated using a NanoDrop 1000 spectrophotometer (Thermo Scientific, USA). Subsequently, 0.5 and 0.2 μg of RNAs were electrophoresed on formaldehyde-denaturing 1.2% agarose gel, and RNA transcripts were evaluated by comparing with a single-stranded (ss) RNA ladder (New England Biolabs, USA) and RNA transcripts of known size, and by comparing the intensity of ethidium bromide staining. For inoculation, RNA transcripts were mixed with a buffer containing a final concentration of 0.1 M Tris-HCl, 10 mM EDTA (pH 7.5), and 0.5 mg/mL bentonite. Two leaves per plant in *N. occidentalis* plants at the 4–5 leaf stage grown on a shelf under controlled conditions (16 h fluorescent light and 24 °C temperature) were dusted with carborandom (600 mesh) and rub-inoculated with 0.1–5 μg of RNA transcripts in 2.5 μL of inoculum per plant using a finger covered in a finger cot. Plants of *C. quinoa* grown under a controlled temperature of 25 °C in the greenhouse were rub-inoculated with the sap of *N. occidentalis*, containing PVS generated by inculation with RNA transcripts, using carborandom and cotton swabs. The appearance and development of symptoms on plants were observed and compared with those obtained by inoculation with the original virus.

### ELISA and northern blot hybridization

To detect viral CP, upper leaves of plants inoculated with RNA transcripts or virus were used for double antibody sandwich (DAS)-ELISA [28] approximately 4 weeks post inoculation. Wells of microplates (Nunc-Immunoplate II; Thermo Scientific, USA) were coated with a rabbit polyclonal antibody (1 μg/mL) against purified PVS-H95 particles produced in our laboratory as described [28]. Alkaline phosphatase conjugated polyclonal antibody against PVS (Adgen) was used at a 1:5000 dilution. Hydrolysis of a colorimetric substrate *p*-nitrophenyl phosphate was measured at 405 nm using Wallac 1420 ARVO MX multilabel plate counter (PerkinElmer, USA).

For northern blot hybridization, total RNA was extracted from the upper leaves of *N. occidentalis* 14 or 15 days post inoculation (dpi) using TRIzol Plus RNA Purification Kit (Invitrogen, USA). The isolated total RNA (10 μg) was electrophoresed on formaldehyde-denaturing 1.2% agarose gel, and then transferred onto Amersham Hybond-N nylon membrane (GE Healthcare, UK). The RNA was fixed to the nylon membrane by exposure to ultraviolet light, and hybridized with a digoxigenin (DIG)-cRNA probe (ca. 800 nucleotides) complimentary to the 3′ terminal region of PVS-H00 genome, excluding the last 9 nucleotides and poly(A) tail at the 3′ end. RNA hybridization was detected with the DIG detection system and chemiluminescent substrate CDP-Star (Roche, Germany), and visualized using LAS-4000mini luminescent image analyzer (Fujifilm, Japan).

### Sequence analysis

The cloned cDNAs were sequenced using an automated DNA sequencer, Li-Cor Model 4000 L (Li-Cor, USA), with Thermo Sequenase fluorescent labeled primer cycle sequencing kit (Amersham, UK), or ABI PRISM 310 Genetic Analyzer (Applied Biosystem, USA) with BigDye Terminator v1.1 cycle sequencing kit (Applied Biosystem, USA). Primers used for cDNA sequencing are listed in Additional file 1: Table S1. The sequences were analyzed using DNASIS software (Hitachi Software Engineering, Japan). The ratio of nonsynonymous to synonymous substitution rates (Ka/Ks) was determined using KaKs_Calculator 2.0 [29] with the LPB method [30, 31].

## Results

### Non-infectious full-length cDNA clones of PVS-H95

First, a full-length cDNA clone pPVS-H-FL-AB was constructed. Three amino acid substitutions, including Ser44Cys, Ile660Val, and Ser908Pro, due to nucleotide substitutions A192T, A2040G, and T2784C, respectively, were generated in ORF1 (replicase) of pPVS-H-FL-AB compared with the consensus sequence of PVS-H95 genome (Fig. 1). Capped RNA, possessing no additional non-viral sequences, was transcribed from the pPVS-H-FL-AB and a single RNA (ca. 8.5 kb) was confirmed by denaturing gel electrophoresis; however, RNA transcripts failed to infect *N. occidentalis* plants. Next, pPVS-H-FL-AB was modifed to obtain an infectious cDNA clone. pPVS-H-FL-V with Ser-44 and pPVS-H-FL-C with Ile-660 and Ser-908 were constructed by the modification of ORF1; however, capped RNA transcripts derived from the two clones also failed to infect *N. occidentalis*. Then, pPVS-H-FL-D with Arg-128, pPVS-H-FL-E with Asn-21, and pPVS-H-FL-F with Asn-21 and Arg-128 were constructed by the conversion of TGBp1 encoded in ORF2 of pPVS-H-FL-C (Fig. 1). After that, pPVS-H-FL-G was constructed by the conversion to Gly-54 in CP encoded in ORF5 of pPVS-H-FL-D (Fig. 1). Finally, pPVS-H-FL-H, whose sequence was identical to the consensus sequence of PVS-H95 genome, was constructed from pPVS-H-FL-C (Fig. 1). However, all capped RNA transcripts derived from these clones still failed to infect *N. occidentalis*.

### An infectious full-length cDNA clone of PVS-H00

**Fig. 3:**
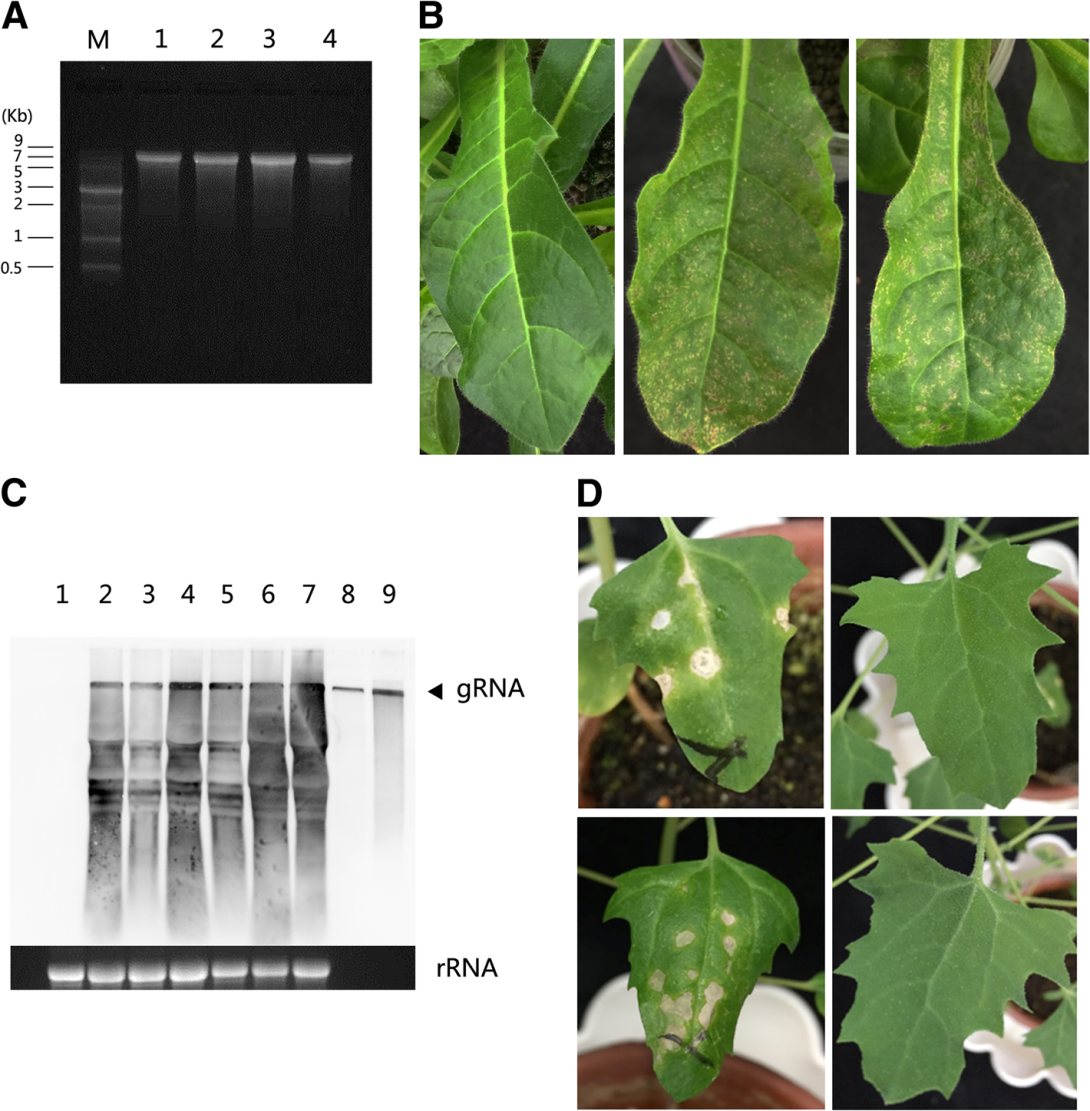
PVS RNA transcription from pPVS-H-FL-β and inoculation of plants. **a** Formaldehyde-denaturing 1.2% agarose gel electrophoresis of RNA transcribed from pPVS-H-FL-β. M: ssRNA marker, lanes 1–4: capped RNA (0.5 μg) transcribed in vitro from pPVS-H-FL-β linearized using *Spe*I restriction endonuclease. **b** Upper leaves of *Nicotiana occidentalis* plants inoculated with mock at 15 days post inoculation (dpi) (left), PVS-H00 at 10 dpi (middle), and capped RNA transcripts from pPVS-H-FL-β at 15 dpi (right). **c** Northern blot hybridization of total RNA extracted from systemically infected leaves of *N. occidentalis* plants 14 or 15 dpi with mock (lane 1) and capped RNA transcripts from pPVS-H-FL-β (lanes 2–5) and PVS-H00 (lanes 6 and 7). Capped RNA transcripts (1 ng; lane 8, 10 ng; lane 9) were used as a positive control. The position of PVS genomic RNA (gRNA) is indicated by an arrowhead on right. The lower panel shows ethidium-bromide stained gel with ribosomal RNA (rRNA) as a loading control from corresponding total RNA samples. **d** *Chenopodium quinoa* plants inoculated with PVS-H00 (top) and the sap of *N. occidentalis* infected with capped RNA transcripts derived from pPVS-H-FL-β (bottom) at 17 dpi. Inoculated leaves are shown in *left*, and upper leaves are shown in *right.*

A full-length cDNA clone pPVS-H-FL-β was reconstructed from the genome of PVS-H00, which was isolated from PVS-H95 populations by repeating a single local lesion isolation in *C. quinoa* three times in 2000. A single RNA of ca. 8.5 kb was confirmed in capped RNA transcripts by denaturing gel electrophoresis (Fig. 3a). The RNA transcripts (2 μg) were mechanically inoculated on *N. occidentalis* plants. The inoculated plants developed mosaic and necrosis symptoms on upper leaves 15 days post inoculation (dpi). Symptom onset in plants inoculated with RNA transcripts was approximately 5 days delayed compared with virus inoculation, implying that the delay was caused by the nature of inoculum, i.e. copies of viral RNA genome or virus particles, because no delay was observed when the sap of *N. occidentalis* infected with RNA transcripts was used as inoculum (data not shown). Except for the delay, no significant difference between PVS-H00 and pPVS-H-FL-β RNA transcript inoculation was observed in symptom development (Fig. 3b). PVS infection in the upper leaves of inoculated plants was verified by the detection of CP with DAS-ELISA using anti-PVS antibody (data not shown), and by northern blot hybridization of subgenomic RNAs with a specific probe complimentary to the 3′ end of the PVS genome (Fig. 3c). The viral genomic and subgenomic RNAs accumulated in plants inoculated with RNA transribed in vitro from pPVS-H-FL-β (Fig. 3c, lanes 2–5), similar to that in plants inoculated with PVS-H00 (Fig. 3c, lanes 6 and 7). To ascertain whether the RNA transcripts prepared from pPVS-H-FL-β exhibited the same biological properties as PVS-H00, *C. quinoa* was inoculated with the sap of PVS-infected *N. occidentalis.* The progeny virus produced from the RNA transcripts induced necrotic spots on inoculated leaves, but not on upper leaves, which was consistent with the symptoms produced by PVS-H00 inoculation (Fig. 3d).

The infectivity of RNA transcripts prepared from pPVS-H-FL-β was further investigated on *N. occidentalis* plants following inoculation with different amounts of RNA transcripts, ranging from 0.1 to 5 μg per plant. All plants inoculated with 0.2–5 μg RNA and 90% of the plants inoculated with 0.1 μg RNA were infected, indicating that RNA transcripts were highly infectious (Table 1). The inoculum dose did not affect the type of symptoms (mosaic and necrosis) but affected the date of onset of symptom development. At least 81.8% of the inoculated *N. occidentalis* plants developed symptoms on systemically infected leaves within 14 dpi, when inoculated with ≥1 μg RNA per plant, while this rate decreased to 50% with ≤0.5 μg RNA per plant. All plants inoculated with ≥1 μg RNA developed symptoms within 16 dpi, while one or two plants inoculated with 0.2 or 0.5 μg RNA developed symptoms 2 days later (data not shown).

**Table 1:** Infectivity of RNA transcripts derived from pPVS-H-FL-β

Amount of transcripts used as inoculum (μg per plant)	Infected/inoculated plants^a^	Infection rates (%)	Plants showing symptoms within 14 days post inoculation (%)	Symptoms
5	22/22	100	81.8	M, N
2	12/12	100	83.3	M, N
1	12/12	100	91.7	M, N
0.5	8/8	100	50	M, N
0.2	8/8	100	37.5	M, N
0.1	9/10	90	40	M, N

^a^Total number of infected/inoculated plants in inoculation experiments two or three times

*M* mosaic, *N* necrosis

In addition to pPVS-H-FL-β under control of T7 promoter, full-length cDNA from pPVS-H-FL-β was cloned downstream of the two copies of the cauliflower mosaic virus 35S promoter. The resulting p35S-PVS-H-FL-β construct proved to be infectious to *N. occidentalis* by rub-inoculation of plasmid DNA linearized with *Spe*I; however, the infection rate was much lower (18.75%) than the RNA transcripts, despite inoculating with 6 μg of plasmid DNA per plant (data not shown).

### Analysis of infectious cDNA clone pPVS-H-FL-β

The sequence of infectious cDNA clone pPVS-H-FL-β, which originated from PVS-H00 genome, was determined and compared with the previously determined consensus sequence of PVS-H95 genome. Both complete genome sequences were 8485 nucleotides long, excluding the poly(A) tail at the 3′ end, and were deposited to the DDBJ nucleotide sequence database under the accession numbers LC375227 (PVS-H95) and LC375228 (PVS-H00). Six ORFs were identified in both PVS genomes: ORF1 (nucleotides 63–5990), ORF2 (6025–6708), ORF3 (6686–7012), ORF4 (6976–7176), ORF5 (7218–8099), and ORF6 (8102–8383) (Figs. 1 and 2). In addition, three MTR motifs, seven HEL motifs, and eight POL motifs [26], four OTU-PRO motifs [32], and P-PRO domain [14] were identified in the replicase encoded by ORF1 in both PVS genomes (Additional file 2: Figure S1).

Surprisingly, numerous sequence polymorphisms were identified between the two PVS genomes (Table 2). A total of 370 nucleotide substitutions were identified, which accounted for 4.4% of the total genome. The nucleotide substitution rate was the highest in ORF1 (*replicase*; 5.4%), followed by ORF3 (*TGBp2*; 4.3%), ORF2 (*TGBp1*; 3.9%), ORF4 (*TGBp3*; 2.0%), and ORF6 (*CRP*; 0.4%), and the lowest in ORF5 (*CP*; 0.3%). No substitutions were detected in the non-coding regions at the 5′ or 3′ termini of the genome, intergenic regions, or overlapping regions of TGB.

**Table 2:** Substitutions between the genomes of PVS isolates H95 and H00

		ORF1		ORF2		ORF3		ORF4		ORF5		ORF6		Total	
		Nt	(%)	Nt	(%)	Nt	(%)	Nt	(%)	Nt	(%)	Nt	(%)	Nt	(%)
Nucleotide substitutions	Ti	260	*81.0*	22	*81.5*	12	*85.7*	4	*100*	3	*100*	1	*100*	302	*81.6*
	Tv	61	*19.0*	5	*18.5*	2	*14.3*	0	*0*	0	*0*	0	*0*	68	*18.4*
	Ti/Tv	4.26		4.40		6.00		–		–		–		4.44	
	S	232	*72.3*	18	*66.7*	13	*92.9*	3	*75*	2	*66.7*	0	*0*	268	*72.4*
	NS	89	*27.7*	9	*33.3*	1	*7.1*	1	*25*	1	*33.3*	1	*100*	102	*27.6*
	Ka/Ks	0.38		0.50		0.08		0.33		0.50		–		0.38	
	Total	321	5.4	27	3.9	14	4.3	4	2.0	3	0.3	1	0.4	370	4.4
		Replicase		TGBp1		TGBp2		TGBp3		CP		CRP		Total	
		Aa	(%)	Aa	(%)	Aa	(%)	Aa	(%)	Aa	(%)	Aa	(%)	Aa	(%)
Amino acids substitutions^a^	0–3	22	*27.5*	2	*28.6*	0	*0*	0	*0*	0	*0*	1	*100*	25	*27.5*
	4	22	*27.5*	0	*0*	0	*0*	0	*0*	1	*100*	0	*0*	23	*25.3*
	5	36	*45.0*	5	*71.4*	1	*100*	1	*100*	0	*0*	0	*0*	43	*47.3*
	Total	80	4.1	7	3.1	1	0.9	1	1.5	1	0.3	1	1.1	91	3.3

Percentage in italic letters indicate the proportion of different substitutions shown in the left column to the total substitutions

^a^Substitutions were evaluated using the Structure-Genetic (SG) scoring system. The SG values range from 0 (most drastic change) to 5 (most frequent substitution)

*Nt* Nucleotides, *Aa* Amino acids, *Ti* Transition, *Tv* Transversion, *S* Synonymous, *NS* Nonsynonymous,

Ka/Ks: The ratio of nonsynonymous to synonymous substitution rate

**Fig. 4:**
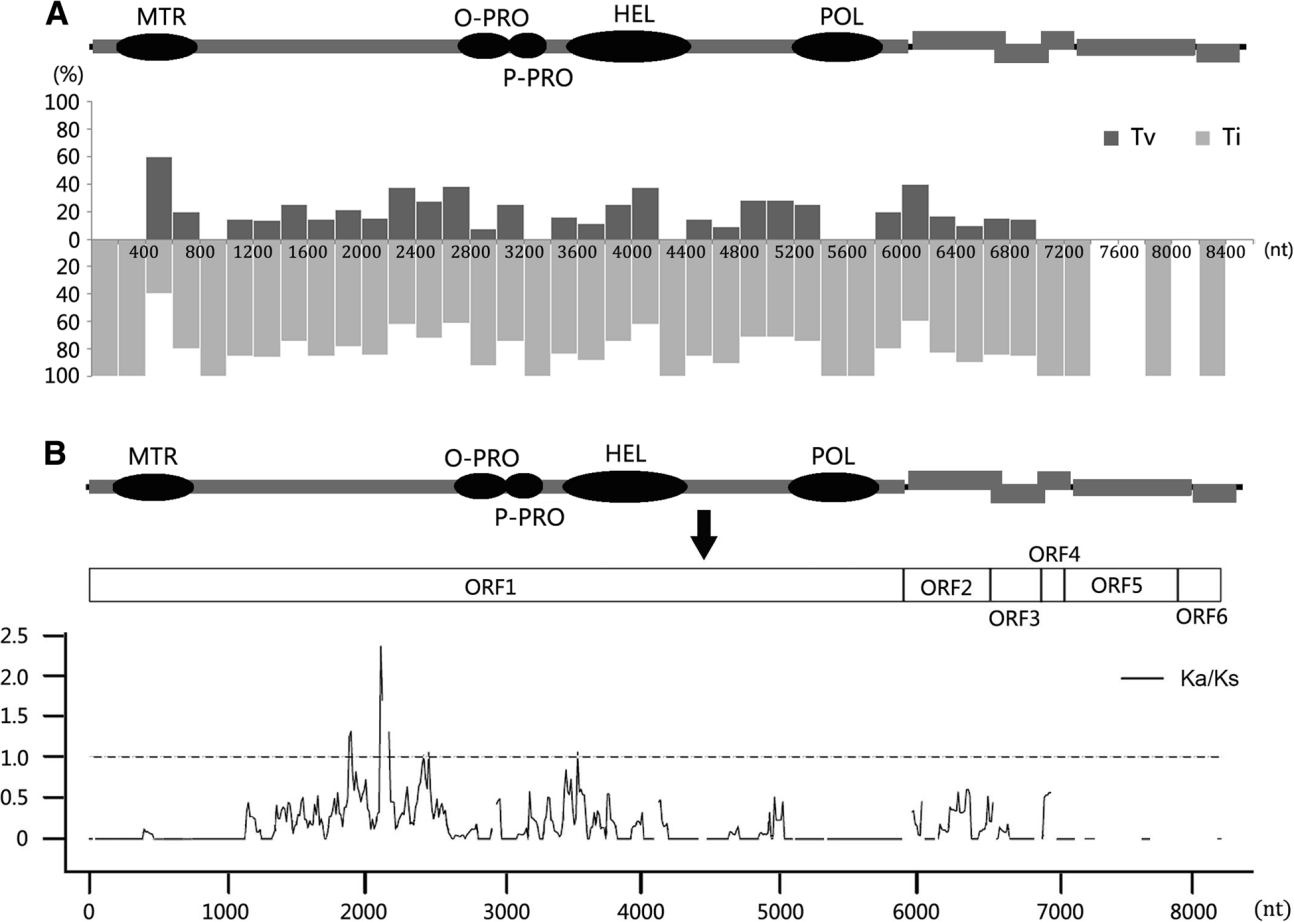
Genome sequence variation between PVS-H00 and PVS-H95. **a** Percentage analysis of transitions (Ti) and transversions (Tv). Percentage of Ti and Tv in each 200-nucleotides (nt) window are shown with light gray bar and dark gray bar, respectively, below the PVS genome map (top diagram). Gray rectangles indicate open reading frames (ORFs), and black ovals represent functional domains in replicase encoded by ORF1. MTR, methyltransferase domain; O-PRO, OTU-like protease region; P-PRO, papain-like cysteine protease region; HEL, helicase domain; POL, RNA-dependent RNA polymease domain. **b** Sliding window analysis of the ratio of nonsynonymous to synonymous nucleotide substitution rates (Ka/Ks) using KaKs_Calculator 2.0. To calculate the Ka/Ks ratio, the full-length genome sequence of PVS (top diagram) was modified to encode a polyprotein created by the seamless connection of six ORFs, i.e. by excluding non-translated and intergenic regions, and duplicating nucleotide sequence of the overlapping region between ORFs (middle diagram). A sliding window profile of Ka/Ks ratio in the modified genome sequences of PVS-H00 and PVS-H95 is shown with a solid line (bottom diagram). Each point plotted represents the Ka/Ks ratio within a sliding window of 75 bp with a step size of 12 bp along the sequence alignment. Ka/Ks ~ 1 (broken line) indicates neutral (i.e. no) selection, > 1 implies positive selection, and < 1 implies negative selection

More than 80% of the nucleotide substitutions in each ORF were transitions (Ti), and transversions (Tv) were detected only in ORFs 1, 2, and 3, and not in ORFs 4, 5, and 6; the Ti/Tv ratio in the entire genome was 4.44 (Table 2). The proportion of Ti and Tv was investigated in every 200-nucleotides intervals in the entire genome (Fig. 4a). The Ti/Tv ratio was the lowest (Ti/Tv = 0.667) in ORF1 from nucleotides 401 to 600, which represented the MTR coding domain.

**Fig. 5:**
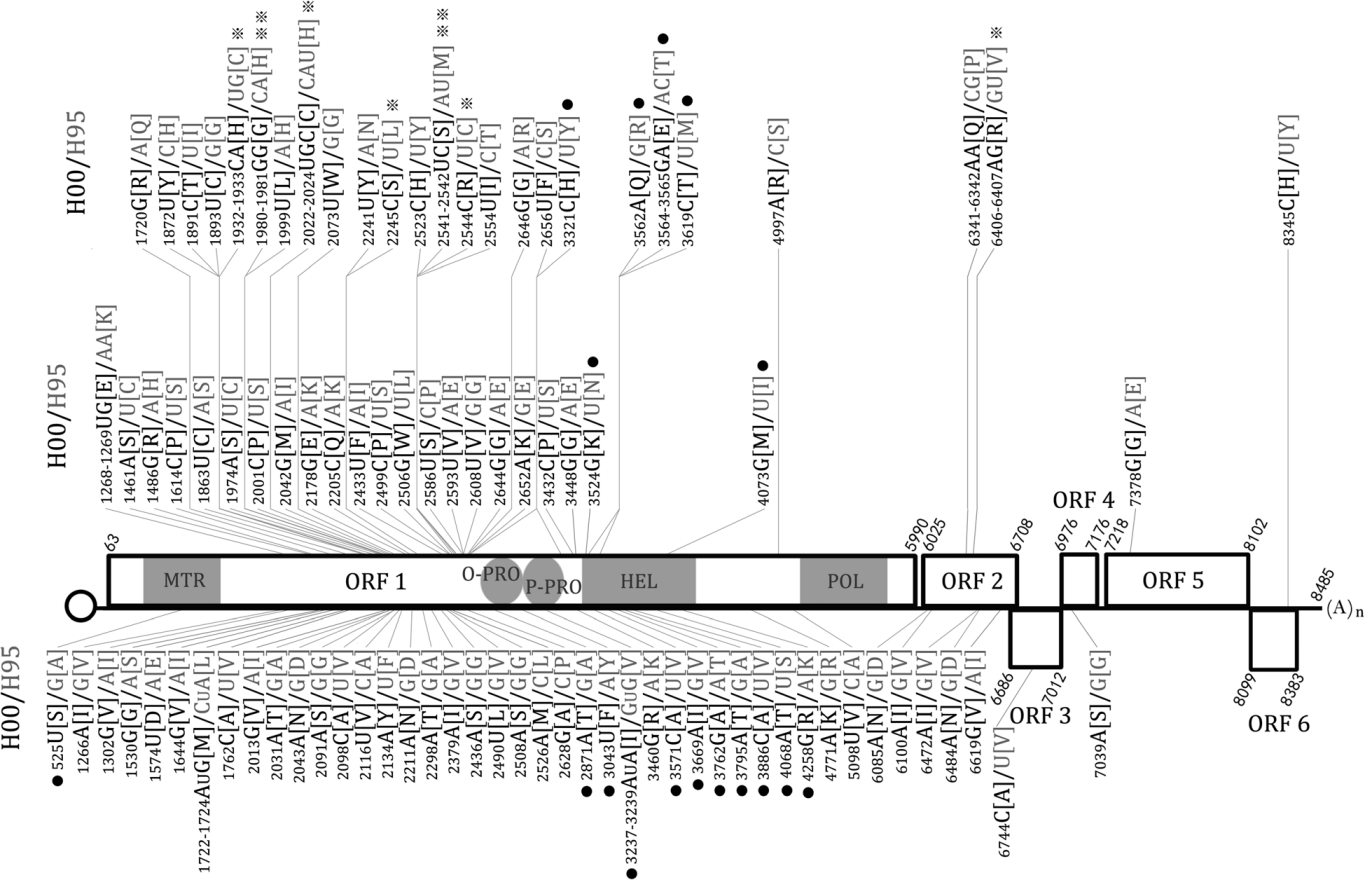
Distribution of amino acid substitutions between PVS-H00 and PVS-H95 genomes. The positions of nucleotide and amino acid substitutions are shown on the PVS genome map. Sequences of PVS-H00 and PVS-H95 genomes are shown in black and gray letters, respectively. Amino acid substitutions were evaluated according to the Structure-Genetic (SG) scoring system. Substitutions in the upper row represent drastic changes (SG = 1–3; ** SG = 1; * SG = 2), those in middle and bottom rows represent relatively similar substitutions (SG = 4), and those in the bottom row represent highly similar substitutions (SG = 5). Substitutions in the functional domains of replicase (ORF1) are indicated with a dot. MTR, methyltransferase domain; O-PRO, OTU-like protease region; P-PRO, papain-like cysteine protease region; HEL, helicase domain; POL, RNA-dependent RNA polymease domain

More than 60% of the substitutions were synonymous. The Ka/Ks ratio is shown in Table 2; Ka/Ks < 1 indicates negative selection on gene sequences, whereas Ka/Ks > 1 indicates positive selection. The Ka/Ks ratio in the entire genome was 0.38, suggesting a strong negative selection on the PVS genome, especially in ORF3 (Ka/Ks = 0.08). However, the Ka/Ks ratio upstream of the OTU-PRO coding region in ORF1 at nucleotides 1885–2247 was > 1.0, with a maximum of 2.37, indicating that this region was under a strong positive selection (Fig. 4b). The Ka/Ks ratio of regions at nucleotides 2425–2535 and 3541–3615 was approximately 1.0, suggesting neutral selection. Nonsynonimous substitutions between PVS-H00 and PVS-H95 genomes amounted to 102 nucleotides, which resulted in 91 amino acid substitutions (Table 2); the distribution of these amino acid substitutions is shown in Fig. 5. Amino acid substitutions showed an uneven distribution among the six ORFs. The rate of amino acid substitution was the highest in replicase (4.1%), followed by TGBp1 (3.1%), TGBp3 (1.5%), CRP (1.1%), and TGBp2 (0.9%), and the lowest in CP (0.3%) (Table 2).

**Fig. 6:**
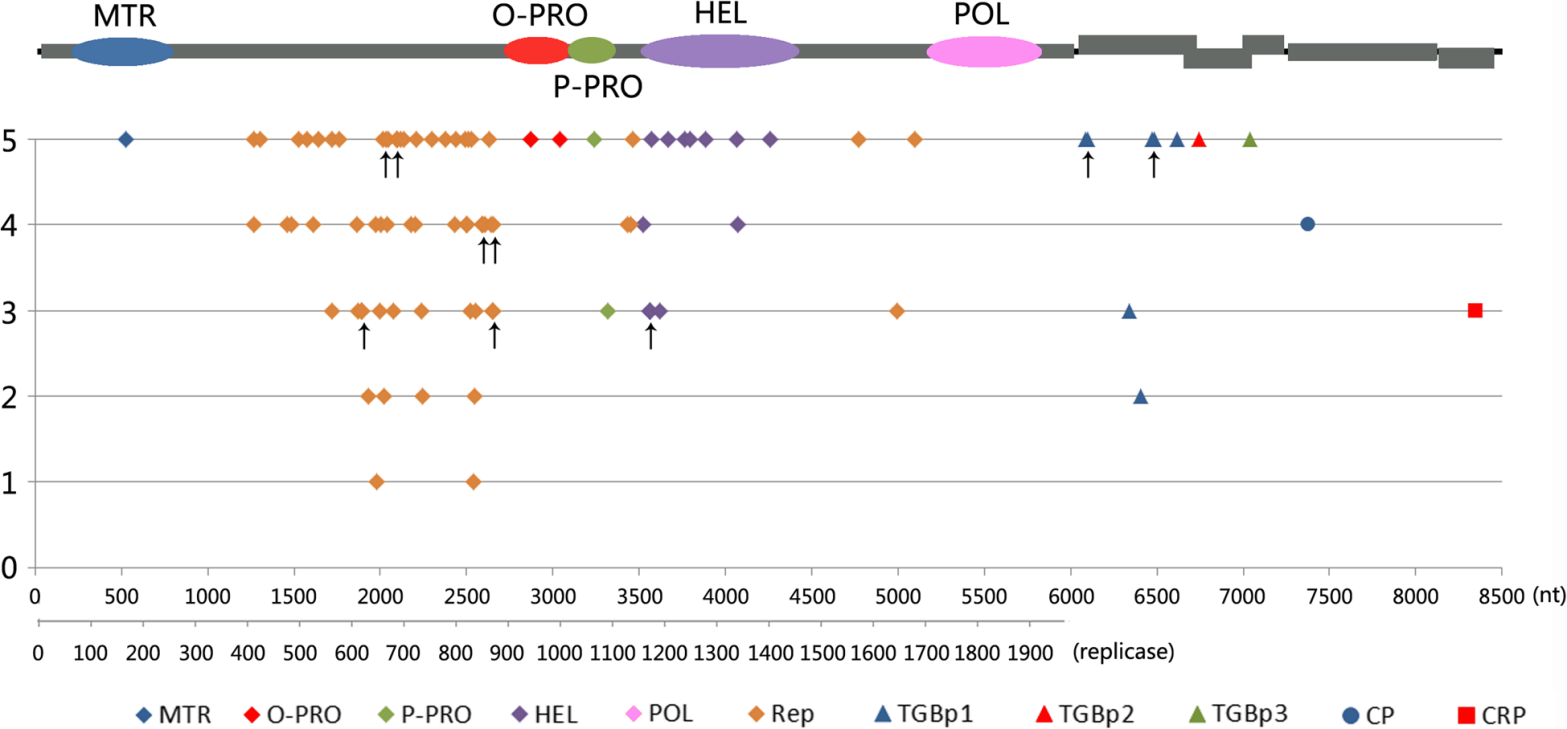
Evaluation of amino acid substitutions in PVS-H00 and PVS-H95 genomes. Each position plotted is based on the evaluation of amino acid substitutions according to the Structure-Genetic (SG) scoring system, and corresponds to the PVS genome map (top diagram). The SG values range from 0 (most drastic change) to 5 (most frequent substitution). MTR: methyltransferase domain; O-PRO: OTU-like superfamily of predicted protease region; P-PRO: papain-like cysteine protease region; HEL: helicase domain; POL: RNA-dependent RNA polymease domain; Rep: other than functional domains within replicase; TGBp1, 2, and 3: triple gene block protein 1, 2, and 3; CP: coat protein; CRP: cysteine-rich protein. Arrows indicate that two or three plots overlap each other

These amino acid substitutions were evaluated using the Structure-Genetic (SG) scoring system, based on structural similarities and interchangeability of amino acids, on a scale of 0–5, where 0 represents the most drastic (rarest) change and 5 represents the most frequent substitution [33] (Table 2, Figs. 5 and 6). Across the entire genome, approximately 50% of the substitutions represented frequent substitutions (SG = 5), approximately 25% represented relatively frequent substitutions (SG = 4), and 27.5% represented drastic changes (SG = 1–3). Amino acid substitutions with SG values of 1–3 were predominant in replicase (27.5%) and TGBp1 (28.6%) with similar proportions, but were not detected in TGBp2, TGBp3, and CP (Table 2). Only one substitution (SG = 3) was detected in CRP (Figs. 5 and 6).

Amino acid substitutions present in replicase, which accounted for approximately 88% of the total substitutions, were further investigated in each functional domain (Figs. 5 and 6, and Additional file 2: Figure S1). The three MTR motifs were identical between the two PVS replicases, except for one substitution Ala155Ser (SG = 5) caused by G525 U nucleotide polymorphism. Two amino acid substitutions detected in the OTU-PRO domain, including Ala937Thr caused by G2871A in motif III and Tyr994Phe caused by A3043U in motif IV, represented frequent substitutions, although Tyr-994 was found only in PVS-H95 among the available PVS genome sequences. Two amino acid substitutions were detected in the P-PRO domain, among which Tyr1087His caused by U3321C represented a relatively drastic change (SG = 3), and Tyr-1087 was identified only in PVS-H95 among the available PVS genome sequences, whereas Val1059Ile caused by GUG3237-3239AUA represented a frequent substitution. The HEL domain harbored more amino acid substitutions than the other four domains including the POL domain, which was identical between the two PVS replicases. In the HEL domain, three substitutions (Arg1167Gln, Thr1168Glu, and Met1186Thr within motif I due to G3562A, AC3564-3565GA, and U3619C, respectively) represented relatively drastic changes (SG = 3), two substitutions (Asn1154Lys and Ile1337Met due to U3524G and U4073G, respectively) represented relatively frequent substitutions (SG = 4), and seven replacements (Val1203Ile within motif IA, Lys1399Arg within motif V, Val1170Ala, Thr1234Ala, Ala1245Thr, Val1275Ala, and Ser1336Thr caused by G3669A, A4258G, U3571C, A3762G, G3795A, U3886C, and U4068A, respectively) represented frequent substitutions (SG = 5). In addition, Thr-1168 was found only in PVS-H95 among the available PVS genome sequences. In replicase, 71.3% of the amino acid substitutions (57/80) were located within the variable region between the MTR and OTU-PRO domains. Among these 57 substitutions, 22 were frequent (SG = 5), 18 replacements were relatively frequent (SG = 4), and the remaining 17 were drastic (SG = 1–3). In replicase, drastic amino acid substitutions with an SG value of 1 were represented by His640Gly and Met827Ser (caused by CA1980-1981GG and AU2541-2542UC nucleotide substitutions, respectively), and those with an SG value of 2 were represented by Cys624His, His654Cys, Leu728Ser, and Cys828Arg (caused by UG1932-1933CA, CAU2022-2024UGC, U2245C, and U2544C, respectively). A Val128Arg substitution (SG = 2) caused by GU6406-6407AG was identified in TGBp1.

### Infectivity of chimeric RNAs derived from recombinants between pPVS-H-FL-β and pPVS-H-FL-H

**Fig. 7:**
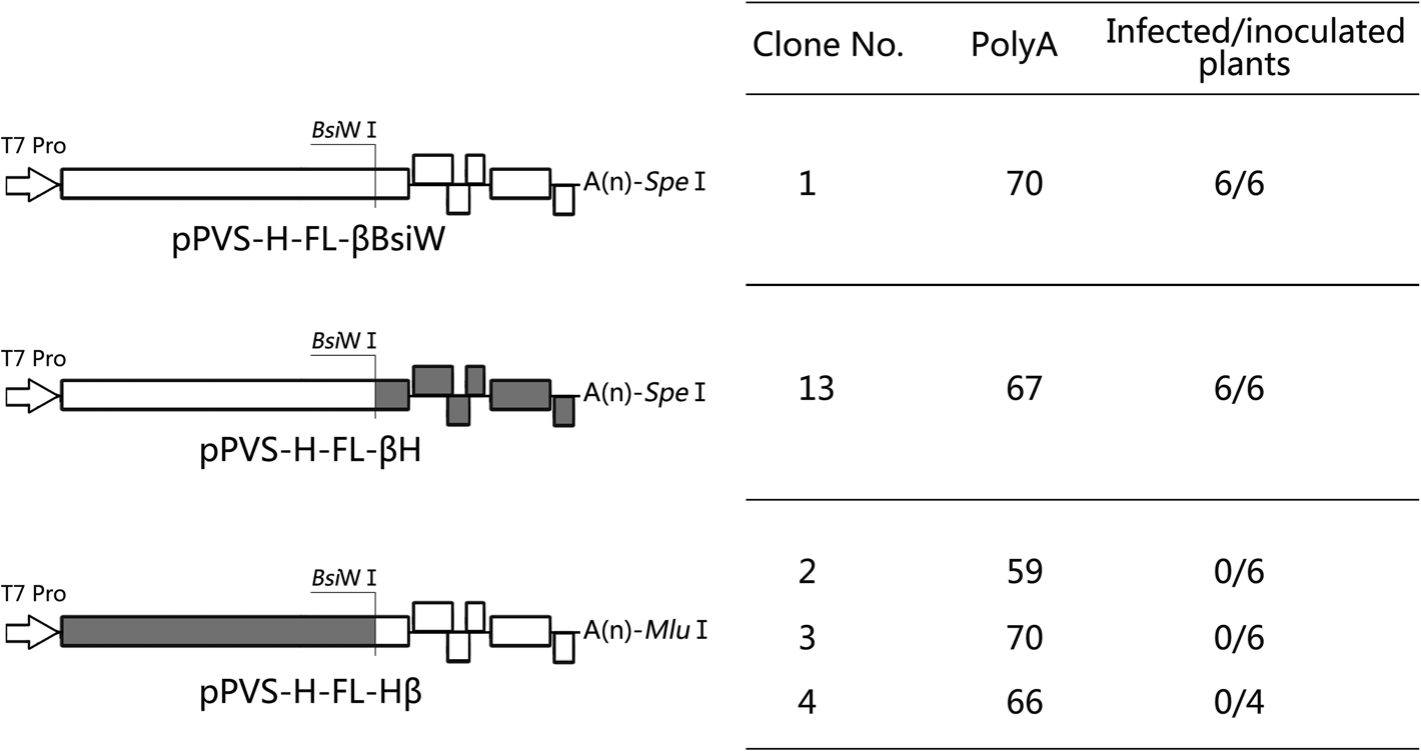
Construction of chimeric cDNA between pPVS-H-FL-β and pPVS-H-FL-H, and the infectivity of RNA transcripts. A unique *Bsi*WI restriction site was introduced by C5850G substitution without an amino acid change by PCR. Two chimeric cDNA clones between pPVS-H-FL-β and pPVS-H-FL-H were constructed by exchanging ORF1 sequence at the *Bsi*WI restriction site. The length of each poly(A) tail was determined by DNA sequence analysis. Capped RNA transcribed from each clone was used to inoculate *N. occidentalis* plants

To investigate whether the lack of infectivity of RNA transcripts derived from pPVS-H-FL-H was caused by amino acid substitutions in replicase or in the other five proteins encoded by the viral genome, we constructed chimeric cDNA clones by exchanging ORF1 sequence between pPVS-H-FL-β and pPVS-H-FL-H (Fig. 7). The exchange was performed by ligation of direct and inverse PCR products at the *Bsi*WI restriction site, which was introduced by the C5850G nucleotide substitution without causing an amino acid change because ORF1 sequence downstream of the *Bsi*WI site was identical between both genomes. A pPVS-H-FL-βBsiW was also generated by inverse PCR to verify the influence of the C5850G substitution. Sequence analyses revealed an unintended alteration in the poly(A) tail length (59–70 adenines in the newly constructed clones compared with 66 adenines in pPVS-H-FL-β) (Fig. 7). The capped RNAs transcribed from pPVS-H-FL-βH and pPVS-H-FL-βBsiW infected *N. occidentalis*, whereas those prepared from the three clones of pPVS-H-FL-Hβ showed no infectivity.

## Discussion

Construction of infectious clones for plant viruses plays a central role in studies of the genome function of viruses and interaction between viruses and host plants by reverse genetics approaches. In this study, we constructed a stable full-length cDNA clone for PVS and an in vitro transcribed RNA with high infectivity to *N. occidentalis*. The process of constructing an infectious full-length cDNA clone of PVS was difficult. Although pPVS-H-FL-H contained sequence that was identical to the determined consensus sequence of PVS-H95 genome, RNA transcripts from pPVS-H-FL-H showed no infectivity, similar to the other seven RNA transcripts harboring 1–4 nucleotide substitutions, which resulted in 1–3 amino acid substitutions. In the host, RNA virus populations are assumed to be essentially a mixture of sequence variants, whose genomes are closely related but unidentical, known as quasispecies (reviewed in [34]). It was assumed that the consensus sequence of PVS-H95, which was determined from at least three clones of each cDNA of five genomic regions (5′ RACE clone, 3′ RACE clone, pPVS-11P11M, pPVS-3P4M, and pPVS1P1M), was a chimeric sequence of discontinuous genome from quasispecies populations.

Thereafter, we attempted to generate a full-length cDNA clone divided into two large overlapping fragments amplified by RT-PCR. The 3′ terminal sequence was replaced by a fragment in which a unique *Spe*I restriction site was created immediately after the poly(A) tail of 66 adenines. Additionally, we used the genome of a pure isolate PVS-H00, selected from PVS-H95 populations by repeating a single local lesion isolation in *C. quinoa* three times, as a template for the RT-PCR. The chlorotic local lesion induced in *C. quinoa* and *C. amaranticolor* by rub-inoculation with PVS is considered to be caused by a hypersensitive response (HR), which is one of the most notable resistance responses. The single local lesion isolation of plant virus in a host plant is considered as a genetic bottleneck, which leads to a reduction in genetic variation in the viral population [35]. In the local lesion cells of *N. glutinosa* infected with tobacco mosaic virus, approximately 10^3^ virus particles were present per cell, which is two to four orders magnitude lower than that in systemically infected cells [36]. The HR and induction of cell death in response to cucumber mosaic virus infection in *C. amaranticolor* has been shown to require cell-to-cell movement of the virus via plasmodesmata outside an initially infected cell [37]. The cell-to-cell movement of plant virus is also considered as one of the genetic bottlenecks in a local lesion host *C. quinoa* inoculated with the soil-borne wheat mosaic virus [38]. Thus, in this study, the population size of PVS-H00 was expected to become much smaller than that of PVS-H95 because of bottleneck events, leading to drastically low genetic variation. The serial local lesion transfer of plant virus has been reported to cause fitness decline [35]. However, Miyashita and Kishino [38] suggest that plant RNA viruses use repeated genetic bottlenecks in each cell-to-cell movement, rather than suffering from fitness loss caused by the bottlenecks, because of a few selected adaptive variants of *trans*-acting genes or elements responding to host shifting and changes in growth conditions. Thus, our data are consistent with Miyashita and Kishino [38] because no biological differences were observed in some host plants between PVS-H00 and PVS-H95. In other words, PVS-H00 may be a selective variant adapted to host plants such as *N. occidentalis* and *C. quinoa*. The existence of PVS-H00 genome in PVS-H95 populations was partially verified by direct sequencing of RT-PCR products amplified from PVS-H95 genome as a minor signal (Additional file 3: Figure S2).

Surprisingly, 370 nucleotide substitutions (4.4%), resulting in 91 amino acid substitutions (3.3%), were identified between PVS-H95 (consensus) and PVS-H00 (pPVS-H-FL-β) genome squences. Phylogenetic analysis of global PVS isolates based on the complete genome sequence shows that PVS-H95 and PVS-H00 are genetically distinct; however, both isolates cluster with six PVS^O^ isolates (Additional file 4: Figure S3). In addition, 95.6% identity was observed between the complete genome sequences of PVS-H95 and PVS-H00, which was derived from PVS-H95, indicating that PVS population originated from a single potato plant shows the same level of genetic diversity as that exhibited by PVS^O^ isolates. The highest amino acid substitution rate of 4.1% (80 amino acid substitutions) was observed in replicase encoded by ORF1, which occupies ca. 70% of the entire PVS genome length. Experiments on the infectivity of chimeric RNAs derived from recombinants between pPVS-H-FL-H and pPVS-H-FL-β revealed that the lack of infectivity of RNA transcripts derived from pPVS-H-FL-H was due to the ORF1 sequence (Fig. 7). The PVS replicase contains five important functional domains (Fig. 5; Additional file 2: Figure S1). Only one amino acid difference (Ala155Ser) observed in the MTR domain of replicase near the N-terminal end represented a frequent substitution (SG = 5). The amino acid sequence of POL domain located near the C terminus of replicase showed no difference, and it was completely identical between both genomes. Although the MTR and POL domains were conserved, HEL domain was the most diverse among the functional domains of replicase, with 12 amino acid substitutions containing 3 relatively drastic substitutions (SG = 3). Of these three substitutions, Met-1186 and Arg-1167 were present among the available PVS genome sequences, whereas Thr-1168, which was conserved in the three sequenced clones, was found only in PVS-H95. The Tyr-994 in OTU-PRO domain was also found only in PVS-H95; however, of three clones sequenced, two harbored Tyr and one harbored Phe, and the replacement of Tyr994Phe represented a frequent substitution (SG = 5). By contrast, the Tyr1087His substitution in the P-PRO domain represented a relatively drastic change (SG = 3), and Tyr-1087 was found only in PVS-H95. The P-PRO processes the replicase polyprotein autocatalytically and affects the infectivity of RNA transcripts in BlScV [14]. In addition to five functional domains, the replicase in some carlaviruses possess the AlkB domain, which repairs RNA damage caused by oxidative demethylation in BlScV [39]. The replicases of PVS and PVM have no typical AlkB motifs; therefore, the corresponding region (PVS replicase amino acids 746–835; nucleotides 2298–2567) located on N-terminal side of the OTU-PRO domain in both viruses appears to be non-functional [13, 40]. Four amino acid substitutions with an SG value 1–3, including Met827Ser (SG = 1), Cys828Arg (SG = 2), Tyr821His (SG = 3), and Thr831Ile (SG = 3), were mapped to the non-functional AlkB domain (Fig. 5). Several additional substitutions with an SG value of 1–3 were located on N-terminal side of the non-functional AlkB domain. The non-functional AlkB domain in PVS replicase implies that an alkylation damage to genomic RNA can not be repaired, and base mispairing may occur during RNA replication, resulting in an increased mutation rate. In a previous study, HpLV purified from commercial hop plants was a genetically heterogeneous population or a quasispecies [26], and 17 out of 50 heterogeneous nucleotides resulted in amino acid substitutions, of which 14 amino acid substitutions were mapped to the replicase containing typical AlkB domain motifs. In addition, of these seventeen amino acid substitutions, five, seven, and five substitutions showed an SG value of 3, 4, and 5, respectively, and none with an SG value of 1–2, unlike PVS in this study, in which two substitutions with an SG value of 1 and five with an SG value of 2 were observed.

To investigate the influence of 80 amino acid substitutions observed between the replicase sequences of PVS-H95 and PVS-H00 on the function, the hydrophobicity patterns were analyzed using ProtScale web server (http://web.expasy.org/protscale/) with the scale of Hphob./Kyte & Doolittle (Additional file 5: Figure S4). Compared with the PVS-H00 replicase, the hydrophobicity of the PVS-H95 replicase declined in a region at amino acids 810–878 in close proximity to the OTU-PRO domain (amino acids 891–1000). Hydrophobic region in a protein play a important role for the protein folding and protein-protein interactions by the hydrophobic effect (reviewed in [41]). Hence, the decline of hydrophobicity in this region may cause the change in tertiary structure and affect the function of the PVS-H95 replicase.

Moreover, the secondary structures of replicases were predicted using PredictProtein web server (http://open.predictprotein.org/) (Additional file 6: Figure S5). Comparisons between PVS-H95 and PVS-H00 indicate a drastically different region in amino acids 596–674 (nucleotides 1848–2084) with eight amino acid substitutions of the SG value 1–3, which is contained in the region showing Ka/Ka ratio > 1.0 (nucleotides 1885–2247) (Fig. 4). The formation of two short α-helix was found in the PVS-H95 replicase. However, because the 80 amino acid substitutions may affect the infectivity in various combinations, it is difficult to answer the question of which amino acid(s) encoded in the consensus ORF1 sequence of PVS-H95 causes the lack of infectivity. Further studies are needed in order to identify the amino acid(s) critical for the function of PVS replicase and understand the replication machinary of PVS using the infectious cDNA clone developed in this study hereafter.

## Conclusions

To the best of our knowledge, this is the first report of an infectious cDNA clone of PVS. Highly infectious RNA transcripts derived from the stable full-length cDNA clone of PVS constructed in this study provide a powerful tool for studies on PVS using reverse genetics, for example, to investigate an unknown genome function, a function in the divergent region of replicase, and pathogenicity difference between PVS^O^ and PVS^A^. In addition, the full-length PVS cDNA clone will be useful for the development of PVS-based vectors for gene silencing and expression of foreign proteins in host plants, such as potato, in the future. Our results suggest that PVS population within a plant exists as quasispecies, and the diversity of replicase coding sequence of carlaviruses or other viruses make it difficult to construct an infectious full-length cDNA clone.

## Additional files

Additional file 1: **Table S1.** Primers used for cDNA amplification and sequencing. (PDF 185 kb)

Additional file 2: **Figure S1.** Comparison of amino acid sequences of functional domains within replicase between PVS-H95 and PVS-H00 genomes. (PDF 275 kb)

Additional file 3: **Figure S2.** Direct sequencing of RT-PCR products amplified from PVS-H95 genome. (PDF 138 kb)

Additional file 4: **Figure S3.** Phylogenetic tree calculated by the maximum-likelihood method from complete genome sequences of global PVS isolates. (PDF 77 kb)

Additional file 5: **Figure S4.** Hydropathy plot of PVS-H95 and PVS-H00 replicases. (PDF 190 kb)

Additional file 6: **Figure S5.** Predicted secondary structure of PVS-H95 and PVS-H00 replicases excluding RNA-dependent RNA polymerase domain. (PDF 271 kb)

## Abbreviations

BlScV: Blueberry scorch virus
CP: Coat protein
CRP: Cysteine-rich protein
DIG: Digoxigenin
dpi: Days post inoculation
ELISA: Enzyme-linked immunosorbent assay
HEL: Helicase
HpLV: Hop latent virus
HR: Hypersensitive response
Ka/Ks: The ratio of nonsynonymous to synonymous substitution rates
MTR: Methyltransferase
ORF: Open reading frame
OTU-PRO: Ovarian tumor-like protease
POL: RNA-dependent RNA polymerase
P-PRO: Papain-like cysteine protease
PVM: Potato virus M
PVS: Potato virus S
PVS^A^: Andean strains of PVS
PVS^O^: Ordinary strains of PVS
RT-PCR: Reverse transcription-polymerase chain reaction
SG: Structure-Genetic
TGB: Triple gene block
Ti: Transitions
Tv: Transversions
