# Supplementary material for: Construction and characterization of an infectious cDNA clone of potato virus S developed from selected populations that survived genetic bottlenecks
Source: Virol J. 2019 Feb 6;16:18. doi: 10.1186/s12985-019-1124-x (PMC6364481; doi:10.1186/s12985-019-1124-x)
Supplement: Supplementary file 1 — Table S1. Primers used for cDNA amplification and sequencing. (PDF 185 kb) [file 12985_2019_1124_MOESM1_ESM.pdf]

Table S1. Primers used for cDNA amplification and sequencing.

Primer name	Primer sequence (5′→3′)[a]	Position[b]	Sense[c]
PVS-1P*	TTGAAGCACCTTTAGGAACA	7162–7181	+
PVS-3P*	CTGGGCTCAAAGTTAGGGAAC	5475–5495	+
PVS-6P	GCTCACAAYGCYCACAAGAG	7965–7984	+
PVS-11P*	ATGTGTCGGCTAGCGCCAAGG	199–219	+
PVS-22P*	CAAGCATGTGCCATTGAAAC	4961–4980	+
PVS-37P*	GCTTCTCACATTCAATCGCCTTCG	4330–4353	+
PVS-41P	ATGAGTTCGTGATCAAATTCGGCGCGGCTT	466–495	+
PVS-42P	GTGAGAAGGATAACTTCGCCTGGTTT*A*GCT ACCATGTGTC	166–205	+
PVS-44P	GCTTAACTGTTGCCGAGTATGCTG	1687–1710	+
PVS-48P	ATGGATGTGTTTTTGCAAGTTTTG	6025–6048	+
PVS-49P	CCCAGGTTTCAATTGGCTGTGC	2422–2443	+
PVS-51P*	*AG*AGATTTGGGTTGGGACGTAC	6406–6427	+
PVS-52P*	AATAGTTGTTCATAGTGTCCC	6093–6113	+
PVS-63P*	GAGGTWTC*G*TACGCCTACAAGCTCG	5841–5865	+
PVSCP1P	TTACTGCTGACATCGCTGG	7576–7594	+
PVSORF6P	AAGCGGAGGGCCCGCAGCATTG	8234–8255	+
T7-PVS-H*	CGATTAAT**TAATACGACTCACTATA**GATAA ACACTCCCGAAAATAATTTGACTT	1–29	+
PVS-1M*	GCATGTCCTATTATCACACT	8304–8323	–
PVS-4M*	GTCTCTCCTGAGCTTGTCGG	7236–7254	–
PVS-11M*	AAGGTGCTAGCCTCACCGGAG	5513–5533	–
PVS-14M*	TTATGTTCGGTAAGAGATCACGCAGG	539–564	–
PVS-21M*	TTTGTCCTGTCGACCTTCGCC	5690–5710	–
PVS-37M*	CCAAATCCCTCTTTGAAGCATGGC	4472–4495	–
PVS-38M*	TGGGTGGTATCACCTCAGTTACTC	8374–8397	–
PVS-41M	AGCGGTATAGTTTGTGGTGTCTCTCCTGAG	7244–7273	–
PVS-42M	GACACATGGTAGC*T*AAACCAGGCGAAGTTA TCCTTCTCAC	166–205	–
PVS-43M	CACATCAATCAAACGTGTGTTGCTC	1727–1751	–
PVS-45M	GGCCCTTCTATGTACTCATCAACC	6258–6281	–
PVS-49M	GGATACGTAGATGATGCTCTTCC	3652–3674	–
PVS-51M*	CAAAAGGGAAGCGGTACAC	6387–6405	–
PVS-52M*	GGTTTAT*T*TAAAGTACTACTAACACGCTC	6064–6092	–
PVS-63M*	CGAGCTTGTAGGCGTA*C*GAWACCTC	5841–5865	–
PVS-H-3E-Spe2*	CACCTGCAGGCACTAGTTTTTTTTTT	poly(A)	–

*Primers marked with an asterisk were used to amplify cDNA of the PVS genome.

[a]Mutated sites in sequences of PVS-42P, -42M, -51P, and -52M primer used for site-directed mutagenesis are indicated in italics.

Nucleotides in bold in the T7-PVS-H primer sequence represent the T7 promoter sequence.

Restriction enzyme recognition sites are underlined.

[b]Binding position within PVS-H95 and PVS-H00 genomes.

[c]Plus (+) or minus (–) indicates homologous and complementary to genomic sequence, respectively.
