# Supplementary material for: Construction and characterization of an infectious cDNA clone of potato virus S developed from selected populations that survived genetic bottlenecks
Source: Virol J. 2019 Feb 6;16:18. doi: 10.1186/s12985-019-1124-x (PMC6364481; doi:10.1186/s12985-019-1124-x)
Supplement: Supplementary file 2 — Figure S1. Comparison of amino acid sequences of functional domains within replicase between PVS-H95 and PVS-H00 genomes. (PDF 275 kb) [file 12985_2019_1124_MOESM2_ESM.pdf]

A

```
                       MTR motif I
PVS-H95        YLSPYSGYPHSHPVCKTLENYLLYKVLPPLVNNTFYFVGIKEFKLNFLKKRIKQMSMIQA   120
PVS-H00        YLSPYSGYPHSHPVCKTLENYLLYKVLPPLVNNTFYFVGIKEFKLNFLKKRIKQMSMIQA   120
               ************************************************************

                 MTR motif II
PVS-H95        INRYVSSADKLRYGNEFVIKFGAASPELKRHHGYALDPALRDLLPNIKRDSNLFFHDEMH   180
PVS-H00        INRYVSSADKLRYGNEFVIKFGAASPELKRHHGYSLDPALRDLLPNIKRDSNLFFHDEMH   180
               ********************************** *************************

                                                             MTR motif III
PVS-H95        YWEKNQLIHFLEQCRPNTCLCTIVYPTEIFVGARRSLNPWAYEFEIKRDKLLFYPDGVRS   240
PVS-H00        YWEKNQLIHFLEQCRPNTCLCTIVYPTEIFVGARRSLNPWAYEFEIKRDKLLFYPDGVRS   240
               ************************************************************

               MTR motif III
PVS-H95        EGYEQPVNCGYLLRTRKILLRDGTMYSVDLVCSKFAHHLIAITKGDLITPTYRSFGPFEA   300
PVS-H00        EGYEQPVNCGYLLRTRKILLRDGTMYSVDLVCSKFAHHLIAITKGDLITPTYRSFGPFEA   300
               ************
```

B

```
                                                              O-PRO motif I
PVS-H95        DPVENAAVGQIATPAPTAGAERSESNEHDAHHTREGVAVHASGKCPAAKKFHRVPNAGGG   900
PVS-H00        DSVVNAAVVQIATPAATAGAGGSKFNEHDAHHTREGVAVHASGKCPAAKKFHRVPNAGGG   900
                                                                  **********

          O-PRO motif I    O-PRO motif II           O-PRO motif III
PVS-H95        DCFWLAISHFTGVSVQDMKQGLQQLEWESDAFSAELALQLKPQAWAEEEAIIATSKQYRY   960
PVS-H00        DCFWLAISHFTGVSVQDMKQGLQQLEWESDAFSAELTLQLKPQAWAEEEAIIATSKQYRY   960
               ************************************ ***********************

         O-PRO motif III               O-PRO motif IV
PVS-H95        RIVVLSADKEQTVIYSPKCEAVQSMVLYHAGAHYEAALPRNDCVLVAVASVLRRRVEEVL   1020
PVS-H00        RIVVLSADKEQTVIYSPKCEAVQSMVLYHAGAHFEAALPRNDCVLVAVASVLRRRVEEVL   1020
               ********************************* ******
```

C

```
PVS-H95        RIVVLSADKEQTVIYSPKCEAVQSMVLYHAGAHYEAALPRNDCVLVAVASVLRRRVEEVL   1020
PVS-H00        RIVVLSADKEQTVIYSPKCEAVQSMVLYHAGAHFEAALPRNDCVLVAVASVLRRRVEEVL   1020
                                                      **********************

PVS-H95        SILGAQLGNEFLQDVLKGEGINRDKLAVVFKLFDICAHVHAEGEVFVINSEGRLHGTFNL   1080
PVS-H00        SILGAQLGNEFLQDVLKGEGINRDKLAVVFKLFDICAHIHAEGEVFVINSEGRLHGTFNL   1080
               ************************************** *********************

PVS-H95        SKDHIEYCKSKPMGITKFTSVHDASCEIKQETLAMLKAMCTLLSYNPCELRAKVLADSLN   1140
PVS-H00        SKDHIEHCKSKPMGITKFTSVHDASCEIKQETLAMLKAMCTLLPYNPCGLRARVLADSLN   1140
               ****** **
```

Figure S1 Comparison of amino acid sequences of functional domains within replicase between PVS-H95 and PVS-H00 genomes. (A) Methyltransferase (MTR) domain; (B) OTU-like protease (O-PRO) region; (C) papain-like cysteine protease (P-PRO) region. Comparative sequences of all domains are shown with flanking sequences. Motifs within each domain are underlined. Identical amino acid residues are indicated with asterisks. Amino acid substitutions with different properties are shown in bold. Amino acid residues identified only in PVS-H95 among all available PVS sequences are highlighted in gray. (*continued*)

D

```
                                                  HEL motif Ⅰ           HEL motif ⅠA
PVS-H95    AGSTGVLCDELFNNVGNLLEANEGRLRTNVREVGCLLGTFGAGKSMVFRKVLSSNLGKSI   1200
PVS-H00    AGSTGVLCDELFNKVGNLLEANEGRLQENAREVGCLLGTFGAGKSTVFRKVLSSNLGKSI   1200
                ******** ************  * *************** **************

           HEL motif ⅠA                                     HEL motif Ⅱ
PVS-H95    IYVSPRKHLADSFNELVKSIKQQEGAASVQGFRTFTFERALLKSAQFRPDATIIIDEIQL   1260
PVS-H00    IYISPRKHLADSFNELVKSIKQQEGAASVQGFRAFTFERALLKSTQFRPDATIIIDEIQL   1260
           ** ****************************** ********** ***************

                             HEL motif Ⅲ                     HEL motif Ⅳ
PVS-H95    FPPGYLDLFSMLAPVGVHMFLVGDPCQSDYDSEKDRSLFQAMKSDINLLLDDADYDFNCR   1320
PVS-H00    FPPGYLDLFSMLAPAGVHMFLVGDPCQSDYDSEKDRSLFQAMKSDINLLLDDADYDFNCR   1320
           ************** *********************************************

        HEL motif Ⅳ
PVS-H95    SRRFKDKLFDGRLPCSIGPMEGEPSKFTIIEGIENCKAIHSQAEVCLVSSFDEKKIVQTY   1380
PVS-H00    SRRFKDKLFDGRLPCTMGPMEGEPSKFTIIEGIENCKAIHSQAEVCLVSSFDEKKIVQTY   1380
           ***************  *******************************************

                  HEL motif Ⅴ               HEL motif Ⅵ
PVS-H95    FPSSCHCFTFGESTGMTYKSGVILITDTSQYTSERRWLTALSRFSHSIAFVNATGGNIQL   1440
PVS-H00    FPSSCHCFTFGESTGMTYRSGVILITDTSQYTSERRWLTALSRFSHSIAFVNATGGNIQL   1440
           ****************** *****************************************
```

E

```
             POL motif Ⅰ             POL motif Ⅱ                POL motif Ⅲ
PVS-H95    MGMVFSKSQLCTKFDNRFRDAKAAQTIVCFQHSVLCRFAPYMRYIEKKLNEVLPARFYIH   1740
PVS-H00    MGMVFSKSQLCTKFDNRFRDAKAAQTIVCFQHSVLCRFAPYMRYIEKKLNEVLPARFYIH   1740
               ********************************************************

           POL motif Ⅲ         POL motif Ⅳ
PVS-H95    SGKGLEELNKWVIESKFDGLCTESDYEAFDASQDQYIVAFELALMRYLGLPNDLIEDYKY   1800
PVS-H00    SGKGLEELNKWVIESKFDGLCTESDYEAFDASQDQYIVAFELALMRYLGLPNDLIEDYKY   1800
           ************************************************************

                                  POL motif Ⅴ            POL motif Ⅵ
PVS-H95    IKTHLGSKLGNFAIMRFSGEASTFLFNTMANMLFTFLRYKLKGDERICFAGDDMCANRAL   1860
PVS-H00    IKTHLGSKLGNFAIMRFSGEASTFLFNTMANMLFTFLRYKLKGDERICFAGDDMCANRAL   1860
           ************************************************************

                                  POL motif Ⅶ         POL motif Ⅷ
PVS-H95    FIKDTHEGFLKKLKLKAKVDRTNRPSFCGWSLCSDGIYKKPQLVFERLCIAKETANLANC   1920
PVS-H00    FIKDTHEGFLKKLKLKAKVDRTNRPSFCGWSLCSDGIYKKPQLVFERLCIAKETANLANC   1920
           ****************************************************
```

Figure S1. (*continued*) Comparison of amino acid sequences of functional domains within replicase between PVS-H95 and PVS-H00 genomes. (D) Helicase (HEL) domain; (E) RNA-dependent RNA polymease (POL) domain. Comparative sequences of all domains are shown with flanking sequences. Motifs within each domain are underlined. Identical amino acid residues are indicated with asterisks. Amino acid substitutions with different properties are shown in bold. Amino acid residues identified only in PVS-H95 among all available PVS sequences are highlighted in gray.
