# Supplementary material for: Construction and characterization of an infectious cDNA clone of potato virus S developed from selected populations that survived genetic bottlenecks
Source: Virol J. 2019 Feb 6;16:18. doi: 10.1186/s12985-019-1124-x (PMC6364481; doi:10.1186/s12985-019-1124-x)
Supplement: Supplementary file 3 — Figure S2. Direct sequencing of RT-PCR products amplified from PVS-H95 genome. (PDF 138 kb) [file 12985_2019_1124_MOESM3_ESM.pdf]

Nucleotide No.	Nucleotide substitutions H95/H00	Amino acids substitutions H95/H00	Direct sequencing of RT-PCR products amplified from PVS-H95 genome
525	G / U	Ala / Ser	T A T G C A C T G G 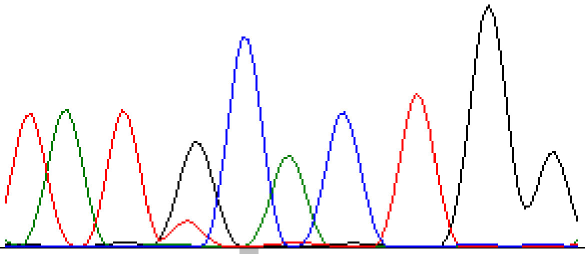
1720	A / G	Gln / Arg	G C A C A A C T A G T 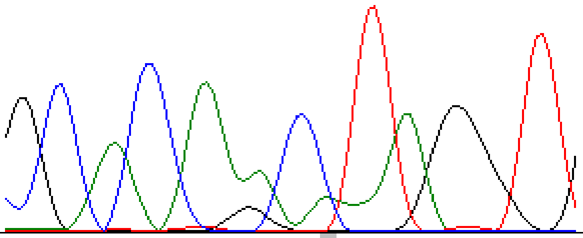
1762	U / C	Val / Ala	T A C T G T T G G 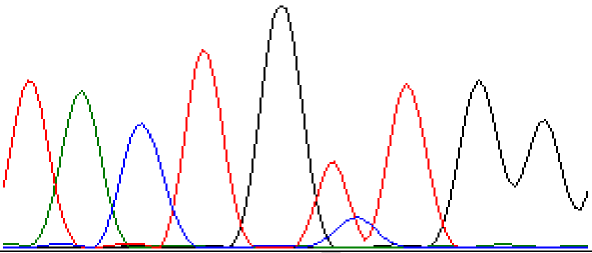
1863	A / U	Ser / Cys	T C G A G A G C G C 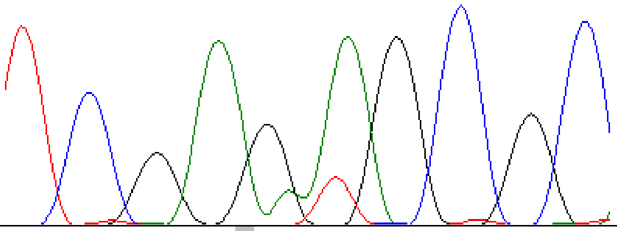
1872	C / U	His / Tyr	A A C A C A T G C A 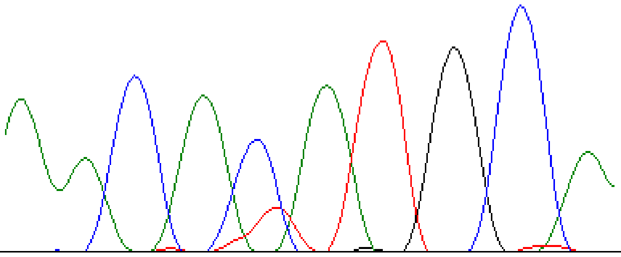

Figure S2 Direct sequencing of RT-PCR products amplified from PVS-H95 genome. Basically, sequence data of nonsynonymous nucleotide substitutions between the genomes of PVS-H95 and PVS-H00 are presented. Double signals are observed in nonsynonymous (shown with gray background) and synonymous (underlined) substitutions, in the sequence data. (*continued*)

Nucleotide No.	Nucleotide substitutions H95/H00	Amino acids substitutions H95/H00	Direct sequencing of RT-PCR products amplified from PVS-H95 genome
1891	U / C	Ile / Thr	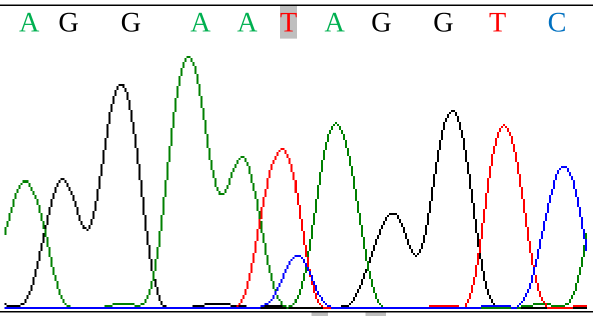
1932-1933	UG / CA	Cys / His	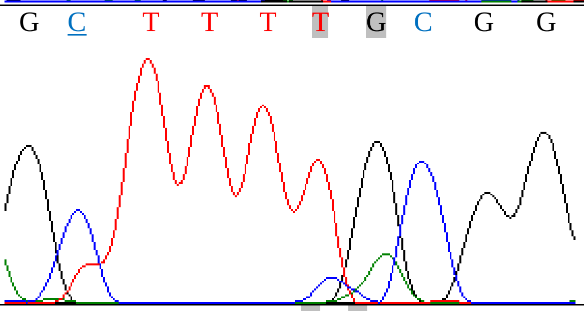
1980-1981	CA / GG	His / Gly	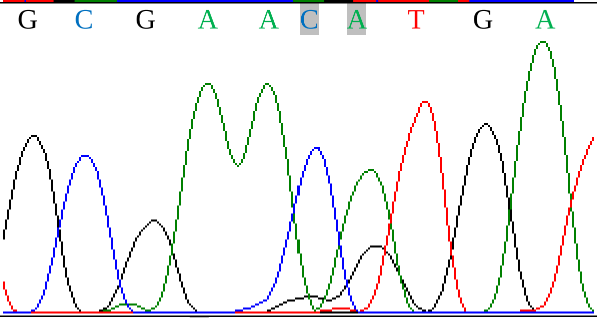
1999	A / U	His / Leu	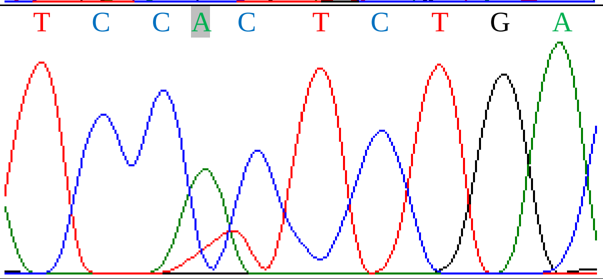
2013	A / G	Ile / Val	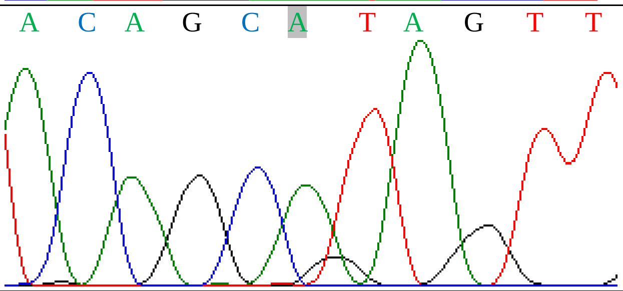

Figure S2 (*continued*) Direct sequencing of RT-PCR products amplified from PVS-H95 genome.

Nucleotide No.	Nucleotide substitutions H95/H00	Amino acids substitutions H95/H00	Direct sequencing of RT-PCR products amplified from PVS-H95 genome
2022-2024	CA / UG	His / Cys	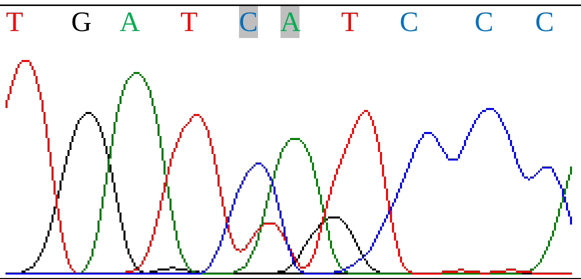
2073	G / U	Gly / Trp	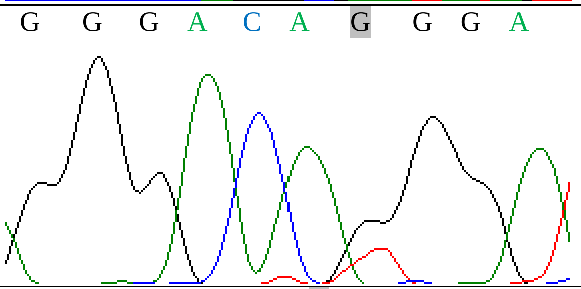
2091	G / A	Gly / Ser	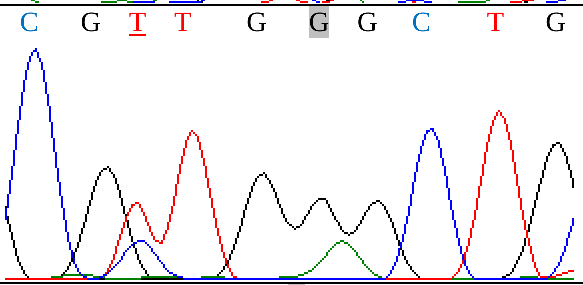
2098	U / C	Val / Ala	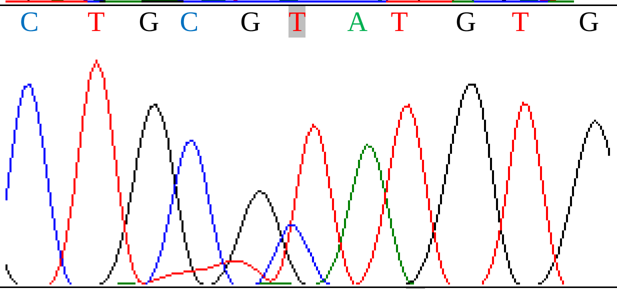
2116	C / U	Ala / Val	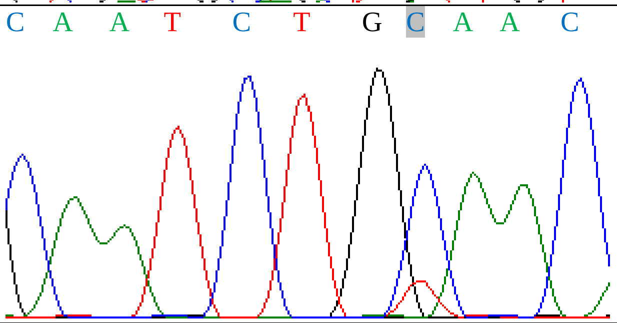

Figure S2 (*continued*) Direct sequencing of RT-PCR products amplified from PVS-H95 genome.

Nucleotide No.	Nucleotide substitutions H95/H00	Amino acids substitutions H95/H00	Direct sequencing of RT-PCR products amplified from PVS-H95 genome
2134	U / A	Phe / Tyr	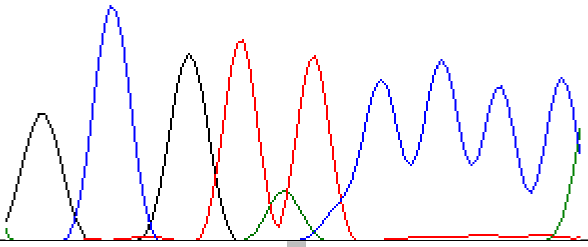
2178	A / G	Lys / Glu	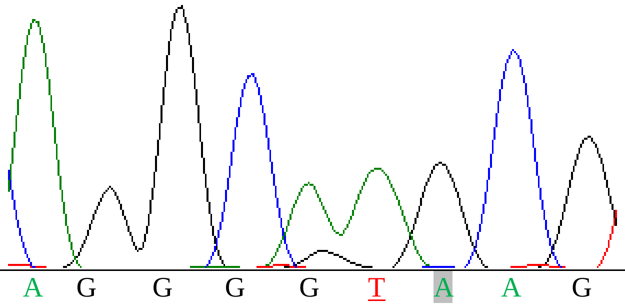
2205	A / C	Lys / Gln	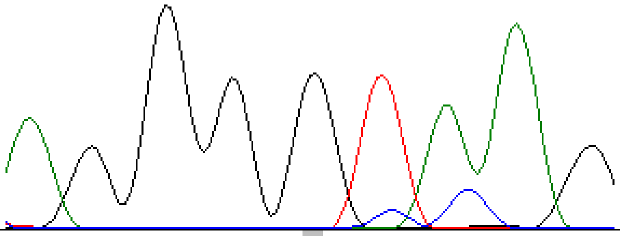
2211	G / A	Asp / Asn	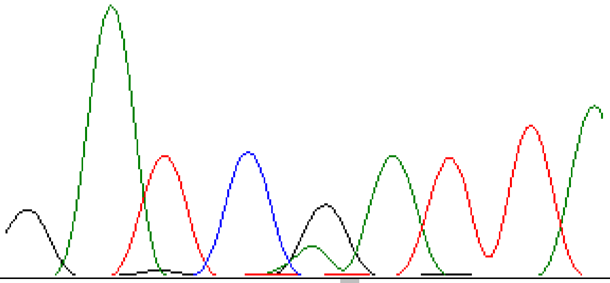
2241	A / U	Asn / Tyr	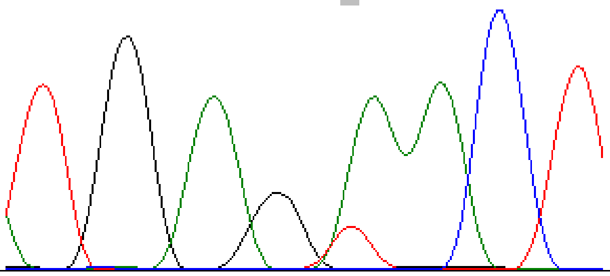

Figure S2 (*continued*) Direct sequencing of RT-PCR products amplified from PVS-H95 genome.

Nucleotide No.	Nucleotide substitutions H95/H00	Amino acids substitutions H95/H00	Direct sequencing of RT-PCR products amplified from PVS-H95 genome
2245	U / C	Leu / Ser	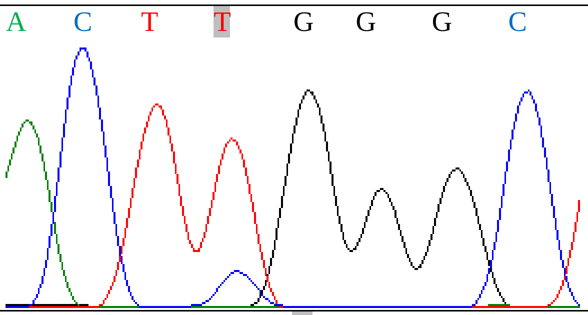
2298	G / A	Ala / Thr	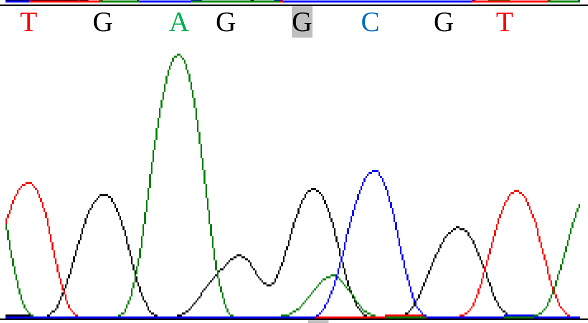
2379	G / A	Val / Ile	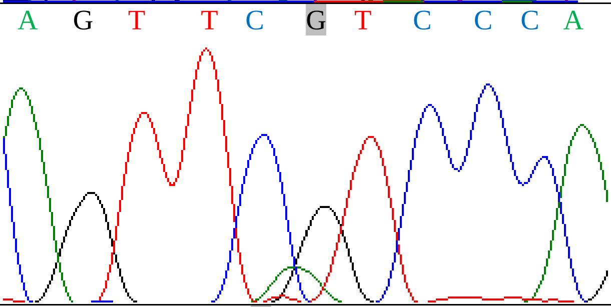
2436	G / A	Gly / Ser	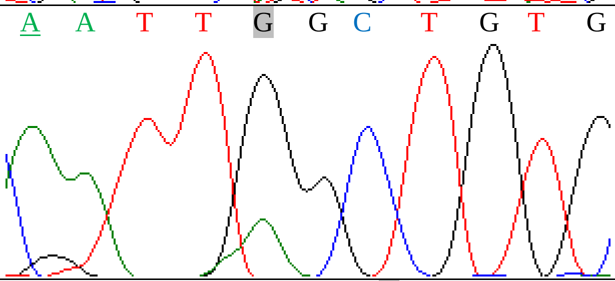
2490	G / U	Val / Leu	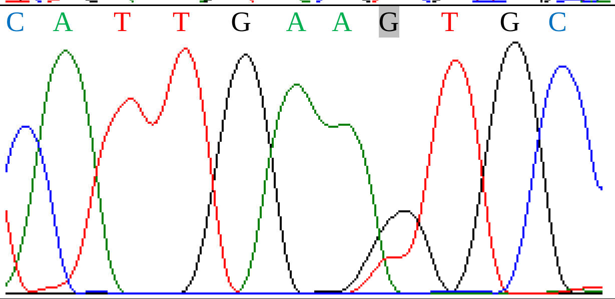

Figure S2 (*continued*) Direct sequencing of RT-PCR products amplified from PVS-H95 genome.
