# Supplementary material for: Construction and characterization of an infectious cDNA clone of potato virus S developed from selected populations that survived genetic bottlenecks
Source: Virol J. 2019 Feb 6;16:18. doi: 10.1186/s12985-019-1124-x (PMC6364481; doi:10.1186/s12985-019-1124-x)
Supplement: Supplementary file 4 — Figure S3. Phylogenetic tree calculated by the maximum-likelihood method from complete genome sequences of global PVS isolates. (PDF 77 kb) [file 12985_2019_1124_MOESM4_ESM.pdf]

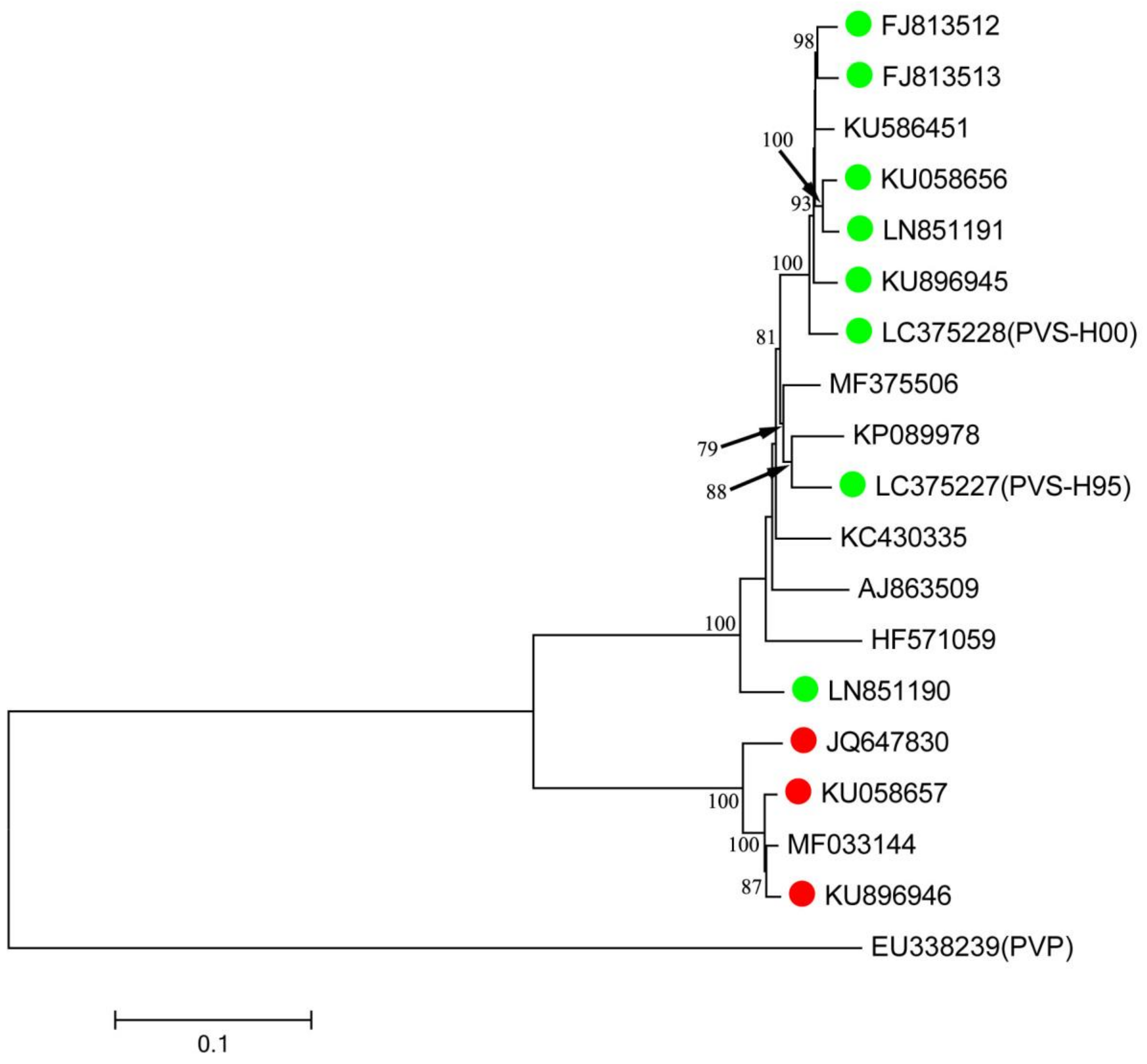

Figure S3. Phylogenetic tree calculated by the maximum-likelihood method from complete genome sequences of global PVS isolates. Phylogenetic relationship of Japanese PVS-H95 and PVS-H00 to global PVS isolates from *S. tuberosum* was examined using MEGA7 software. Sixteen nonrecombinant PVS isolates shown with accession numbers were selected from the DDBJ sequence database, and an isolate of potato virus P (PVP) was used as an outgroup. Biologically PVS^{O} and PVS^{A} isolates are shown with green and red circles, respectively. Biological properties of other isolates are unknown. Bootstrap values (1,000 replicates) of >75% are given at the branch nodes.
