# Supplementary material for: Construction and characterization of an infectious cDNA clone of potato virus S developed from selected populations that survived genetic bottlenecks
Source: Virol J. 2019 Feb 6;16:18. doi: 10.1186/s12985-019-1124-x (PMC6364481; doi:10.1186/s12985-019-1124-x)
Supplement: Supplementary file 5 — Figure S4. Hydropathy plot of PVS-H95 and PVS-H00 replicases. (PDF 190 kb) [file 12985_2019_1124_MOESM5_ESM.pdf]

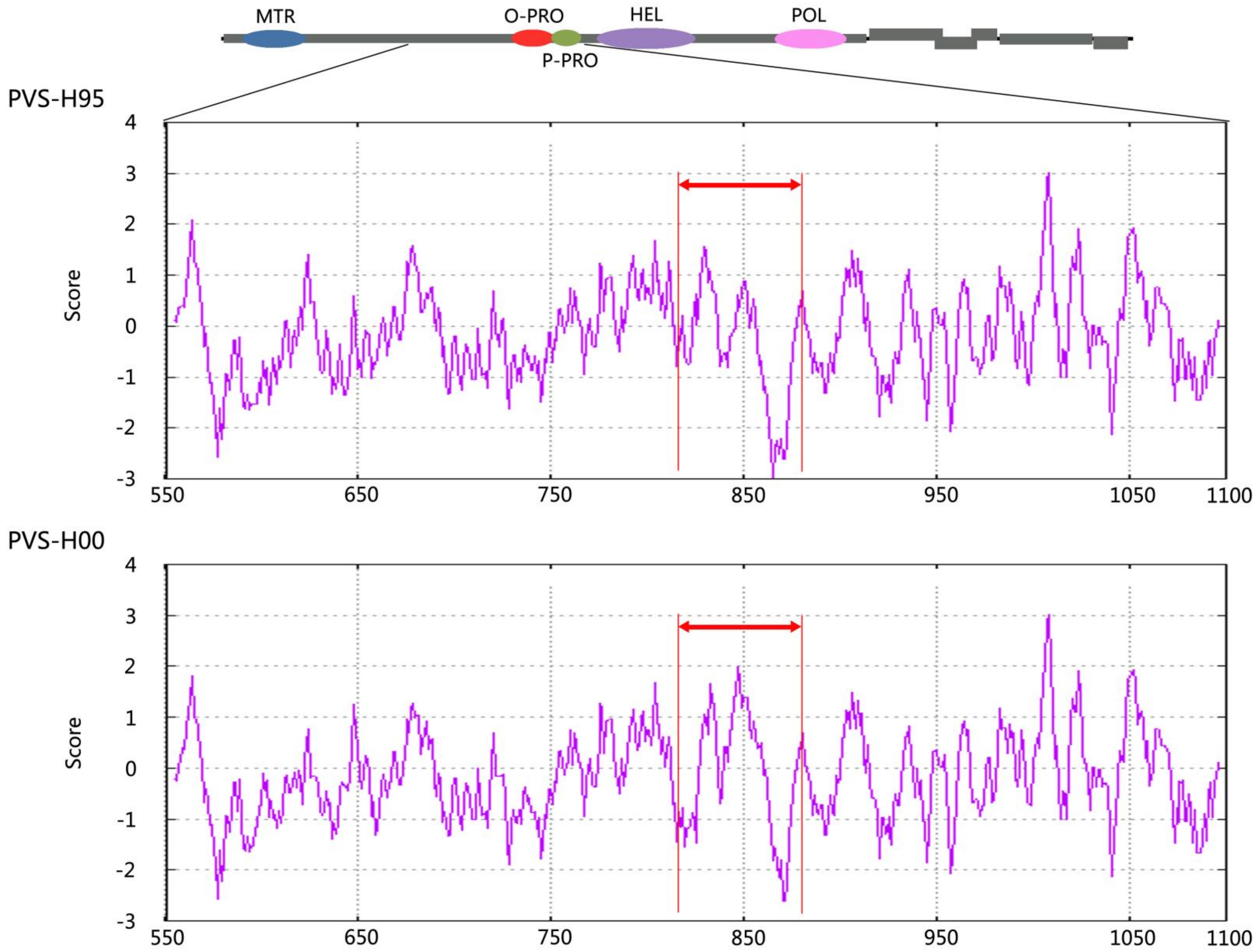
MTR
O-PRO
HEL
POL
P-PRO
PVS-H95
Score
PVS-H00
Score
4
3
2
1
0
-1
-2
-3
550
650
750
850
950
1050
1100

Figure S4. Hydropathy plot of PVS-H95 and PVS-H00 replicases. The hydrophobicity patterns in the replicase amino acids 550-1100, a region indicated on the PVS genomic map at the top, were analyzed using ProtScale web server (http://web.expasy.org/protscale/) with Hphob./Kyte & Doolittle scale. Positive and negative values of score represent the degree of hydrophobicity and hydrophilicity, respectively. Horizontal axis indicates the position of replicase amino acid sequence. Region different between PVS-H95 and PVS-H00 is shown with red double-headed arrows.
