# Supplementary material for: Construction and characterization of an infectious cDNA clone of potato virus S developed from selected populations that survived genetic bottlenecks
Source: Virol J. 2019 Feb 6;16:18. doi: 10.1186/s12985-019-1124-x (PMC6364481; doi:10.1186/s12985-019-1124-x)
Supplement: Supplementary file 6 — Figure S5. Predicted secondary structure of PVS-H95 and PVS-H00 replicases excluding RNA-dependent RNA polymerase domain. (PDF 271 kb) [file 12985_2019_1124_MOESM6_ESM.pdf]

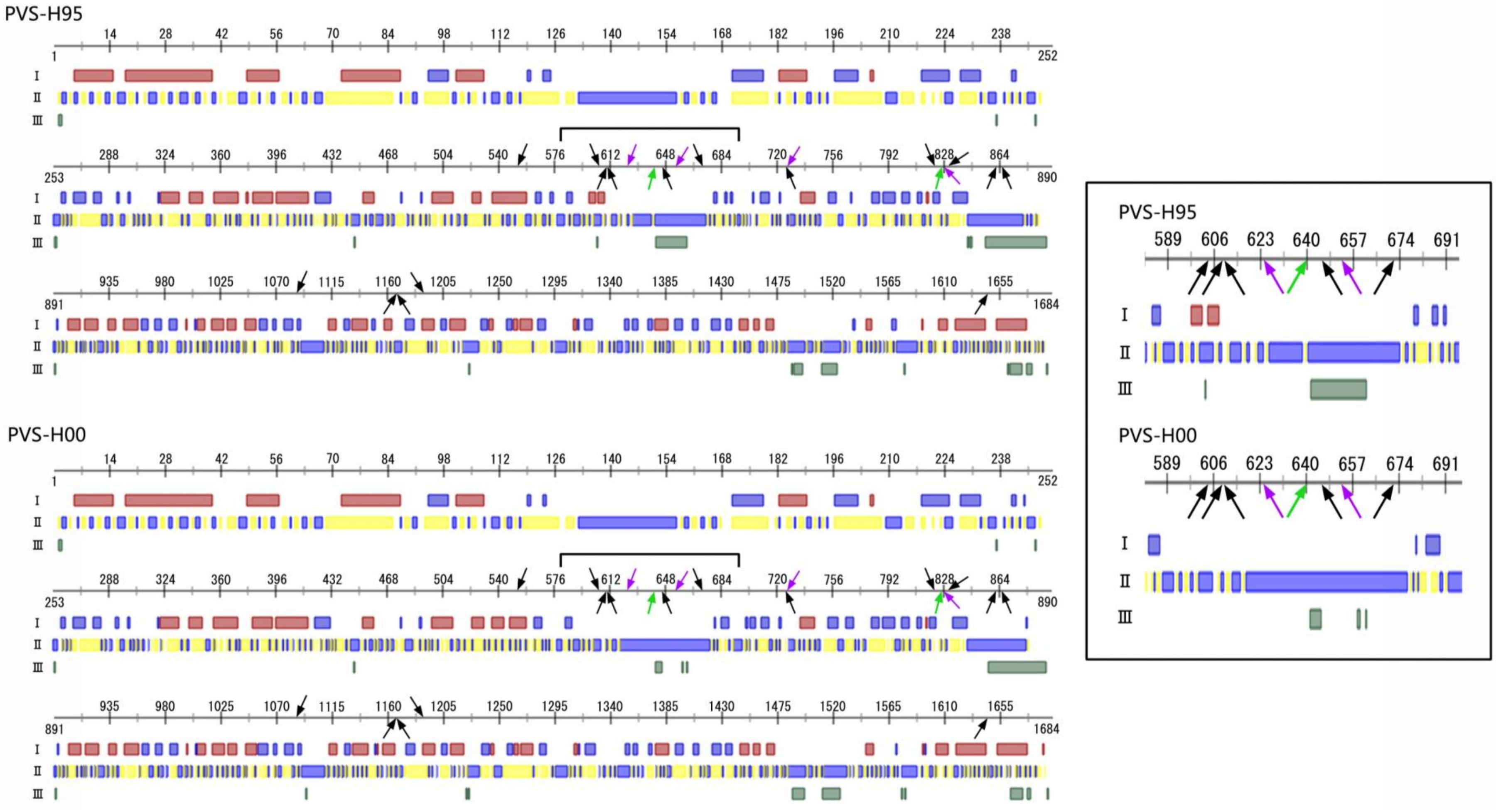
PVS-H95
14 28 42 56 70 84 98 112 126 140 154 168 182 196 210 224 238
1 252
I
II
III
288 324 360 396 432 468 504 540 576 612 648 684 720 756 792 828 864
253 890
935 980 1025 1070 1115 1160 1205 1250 1295 1340 1385 1430 1475 1520 1565 1610 1655
891 1684
PVS-H00
14 28 42 56 70 84 98 112 126 140 154 168 182 196 210 224 238
1 252
288 324 360 396 432 468 504 540 576 612 648 684 720 756 792 828 864
253 890
935 980 1025 1070 1115 1160 1205 1250 1295 1340 1385 1430 1475 1520 1565 1610 1655
891 1684
PVS-H95
589 606 623 640 657 674 691
PVS-H00
589 606 623 640 657 674 691

Figure S5. Predicted secondary structure of PVS-H95 and PVS-H00 replicases excluding RNA-dependent RNA polymerase domain. Protein secondary structures were predicted using PredictProtein web server (https://open.predictprotein.org/). The amino acid sequences of PVS-H95 and PVS-H00 replicases were devided into four parts, and the predicted results presented are three parts because forth RNA-dependent RNA polymerase domain sequences are identical between PVS-H95 and PVS-H00. Replicase sequence numbers are shown at the top line. Structures of α-helix and β-strand are shown with red and blue rectangles, respectively, in line (I). Exposed and buried regions are shown with blue and yellow rectangles, respectively, in line (II). Disordered regions are shown with green rectangles in line (III). The positions of amino acid substitutions between the replicases of PVS-H95 and PVS-H00 are indicated by arrows with 1 (green), 2 (purple) and 3 (black) values evaluated according to the Structure-Genetic (SG) scoring system, as shown in Figs. 5 and 6. Comparisons between PVS-H95 and PVS-H00 indicate a drastically different region shown in vertical left square brackets and enlarged in a right box.
